# Physician Posttraumatic Stress Disorder During COVID-19: A Systematic Review and Meta-Analysis

**DOI:** 10.1001/jamanetworkopen.2024.23316

**Published:** 2024-07-24

**Authors:** Mihir Kamra, Shan Dhaliwal, Wenshan Li, Shrey Acharya, Adrian Wong, Andy Zhu, Jaydev Vemulakonda, Janet Wilson, Maya Gibb, Courtney Maskerine, Edward Spilg, Peter Tanuseputro, Daniel T. Myran, Marco Solmi, Manish M. Sood

**Affiliations:** 1 McMaster University, Hamilton, Ontario, Canada; 2 Faculty of Medicine, University of Ottawa, Ottawa, Ontario, Canada; 3 The Ottawa Hospital Research Institute, Ottawa, Ontario, Canada; 4 Department of Medicine, The Ottawa Hospital, Ottawa, Ontario, Canada; 5 ICES, Ontario, Canada; 6 Bruyere Research Institute, Ottawa, Ontario, Canada; 7 Clinical Epidemiology Program, Ottawa Hospital Research Institute, Ottawa, Ontario, Canada; 8 Department of Family Medicine, University of Ottawa, Ottawa, Ontario, Canada; 9 School of Epidemiology and Public Health, University of Ottawa, Ottawa, Ontario, Canada; 10 Department of Psychiatry, University of Ottawa, Ottawa, Ontario, Canada; 11 Deptartment of Mental Health, The Ottawa Hospital, Ottawa, Ontario, Canada; 12 Department of Child and Adolescent Psychiatry, Charité Universitätsmedizin, Berlin, Germany

## Abstract

**Importance:**

The COVID-19 pandemic placed many physicians in situations of increased stress and challenging resource allocation decisions. Insight into the prevalence of posttraumatic stress disorder in physicians and its risk factors during the COVID-19 pandemic will guide interventions to prevent its development.

**Objective:**

To determine the prevalence of posttraumatic stress disorder (PTSD) among physicians during the COVID-19 pandemic and examine variations based on factors, such as sex, age, medical specialty, and career stage.

**Data Sources:**

A Preferred Reporting Items for Systematic Reviews and Meta-analyses–compliant systematic review was conducted, searching MEDLINE, Embase, and PsychInfo, from December 2019 to November 2022. Search terms included MeSH (medical subject heading) terms and keywords associated with physicians as the population and PTSD.

**Study Selection:**

Peer-reviewed published studies reporting on PTSD as a probable diagnosis via validated questionnaires or clinician diagnosis were included. The studies were reviewed by 6 reviewers.

**Data Extraction and Synthesis:**

A random-effects meta-analysis was used to pool estimates of PTSD prevalence and calculate odds ratios (ORs) for relevant physician characteristics.

**Main Outcomes and Measures:**

The primary outcome of interest was the prevalence of PTSD in physicians, identified by standardized questionnaires.

**Results:**

Fifty-seven studies with a total of 28 965 participants and 25 countries were included (of those that reported sex: 5917 of 11 239 [52.6%] were male and 5322 of 11 239 [47.4%] were female; of those that reported career stage: 4148 of 11 186 [37.1%] were medical trainees and 7038 of 11 186 [62.9%] were attending physicians). The estimated pooled prevalence of PTSD was 18.3% (95% CI, 15.2%-22.8%; *I^2^* = 97%). Fourteen studies (22.8%) reported sex, and it was found that female physicians were more likely to develop PTSD (OR, 1.93; 95% CI, 1.56-2.39). Of the 10 studies (17.5%) reporting age, younger physicians reported less PTSD. Among the 13 studies (22.8%) reporting specialty, PTSD was most common among emergency department doctors. Among the 16 studies (28.1%) reporting career stage, trainees were more prone to developing PTSD than attendings (OR, 1.33; 95% CI, 1.12-1.57).

**Conclusions and Relevance:**

In this meta-analysis examining PTSD during COVID-19, 18.3% of physicians reported symptoms consistent with PTSD, with a higher risk in female physicians, older physiciansy, and trainees, and with variation by specialty. Targeted interventions to support physician well-being during traumatic events like pandemics are required.

## Key Points

**Question:**

What is the prevalence of posttraumatic stress disorder (PTSD) among physicians during the COVID-19 pandemic, and how does this vary based on factors such as sex?

**Findings:**

In this systematic review and meta-analysis of 57 studies with 28 965 participants, a higher PTSD prevalence among physicians was found compared with the reported literature on the prevalence before the COVID-19 pandemic and the general population. Women and medical trainees were significantly more likely to develop PTSD, and emergency and family medicine specialties tended to report higher prevalence.

**Meaning:**

These findings suggest that physicians were more likely to experience PTSD during the COVID-19 pandemic, which highlights the importance of further research and policy reform to uphold physician wellness practices.

## Teaser

This systematic review and meta-analysis assesses the prevalence and risk factors of posttraumatic stress disorder (PTSD) among physicians during the COVID-19 pandemic.

## Introduction

Physicians are susceptible to developing posttraumatic stress disorder (PTSD). Cohort studies and meta-analyses suggest higher PTSD prevalence in physicians, of more than 15%, compared with the 4% to 5% in the general population.^1,2^ Exposure to patient deaths, life-threatening emergencies, heavy workloads, and workplace violence may contribute to this heightened risk.^3,4,5,6^

The COVID-19 pandemic imposed an unprecedented strain on the health care system. In many regions, the volume of cases, hospitalizations, and fatalities exceeded the capacity that health care systems typically handle. To manage this surge in demand, hospitals extended staff working hours, reduced vacation time, and expanded their intensive care unit capacity.^7,8^ Furthermore, physicians were at high risk for infection themselves. Consequently, many of the postulated risk factors for PTSD among physicians were amplified, consistent with previous community-wide traumatic events that led to significant increases in PTSD among physicians.^9,10,11^ Thus, there is reason to suspect a higher prevalence of PTSD during this pandemic.

The ramifications of PTSD may extend beyond a physician’s well-being and health to their professional capacity and health care delivery or quality. PTSD in physicians is strongly associated with burnout, which has been shown to lead to decreased productivity, compromised patient care, an increased likelihood of medical errors, elevated staff turnover rates, and a heightened risk of suicide.^11,12,13,14,15^ Considering the current global shortage of physicians, the presence of PTSD further exacerbates the strain on the health care workforce, creating a self-perpetuating cycle where the demand for health care services increases, leading to more physician burnout and turnover and subsequently intensifying the demand.

Recognizing the association of COVID-19 with the prevalence of and risk factors for PTSD in physicians will be crucial to inform interventions to shape practices that retain physicians and ensure the continued delivery of high-quality health care services. Thus, we set out to assess the prevalence of PTSD among physicians during the pandemic and explore how it varies with factors such as sex, age, career stage or experience, and specialty. Our findings aim to inform tailored practices to support vulnerable physician populations effectively.

## Methods

This meta-analysis and systematic review followed the Preferred Reporting Items for Systematic Reviews and Meta-analyses (PRISMA) reporting guideline. Institutional review board, ethics committee approval, and informed consent were not needed because data were obtained from existing literature.

This meta-analysis and systematic review followed the Preferred Reporting Items for Systematic Reviews and Meta-analyses (PRISMA) reporting guidelines. MEDLINE, Embase, and PsychInfo were the main databases searched for relevant articles from December 2019 to November 2022. Search terms included MeSH (medical subject heading) terms and keywords associated with physicians as the population and PTSD. Six authors were involved in study selection, data extraction, and risk of bias assessment.

### Protocol

**Figure 1.**
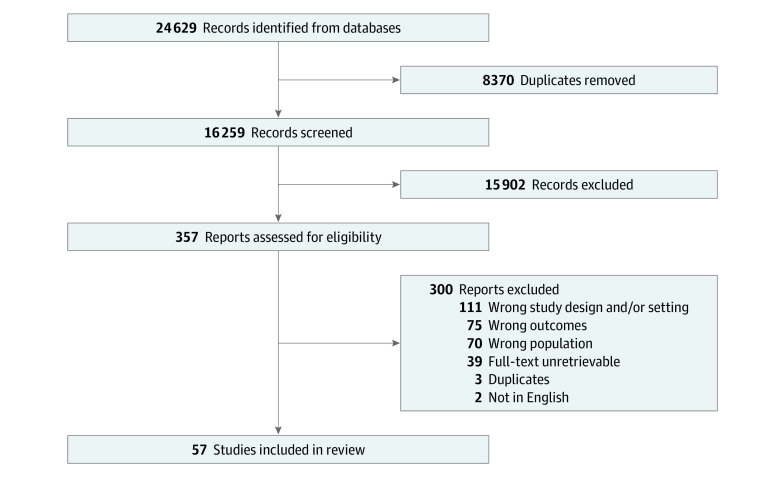
PRISMA Flowchart

A detailed search strategy is presented in eAppendix 1 in Supplement 1. Validated questionnaires include the Impact of Event Scale-Revised (IES-R) and the PTSD Checklist (PCL) for *Diagnostic and Statistical Manual of Mental Disorders* (Fifth Edition) (*DSM-V*) (PCL-5). Scales that were variants of these questionnaires or had adequate evidence of their validity were deemed appropriate to include. Study flow diagram is presented in Figure 1 with exclusion criteria in eAppendix 2 in Supplement 1.

In this study, the primary outcome was the prevalence of PTSD in physicians, identified by standardized questionnaires. Studies that stratified PTSD symptoms by mild, moderate, or severe cases were defined by what the authors of that article deemed to be the appropriate cutoff for PTSD. Further detailed descriptions of the questionnaires, including their structure and what contexts they have been validated, are included in Table 1 and eAppendix 2 in Supplement 1.

**Table 1.** Design, Method of Measurement, and Outcomes for Included Studies Assessing PTSD in Physicians

Source	Location	Study design	Sample, No.	Response rate, %	Outcome assessment	Definition of outcome	Outcome, no. (%)
Wang et al,^17^ 2020	China	CSS	563	73.6	IES-R	Mean score of at least 2.0 across all items (there are 88 items) based on the 4 response options for each item 4 response options (0, not at all; 1, seldom; 3, sometimes; 5, often); cutoff (implied), ≥44	Point prevalence: 39 (6.9)
Moderato et al,^18^ 2021	Australia	CSS	658	NR	IES-R	Severe PTSD symptoms: ≥33	Point prevalence: 236 (35.9)
Udgiri et al,^19^ 2021	India	CSS	80	NR	IES-R	Mild, ≥24; moderate, ≥33; severe, ≥37; moderate considered the best cutoff for probable PTSD diagnosis	Point prevalence: mild, 42 (52.5%); moderate, 16 (20%); severe: 9 (11.3%)
Dykes et al,^20^ 2022	UK	CSS	43	NR	IES-R	Suggestive of PTSD, 12-32; consistent with PSTD, ≥33; probable PTSD diagnosis, ≥33	Point prevalence: suggestive of PTSD, 18 (41.9); consistent with PTSD, 6 (14.0)
Pascoe et al,^21^ 2022	Australia	CSS	1966	NR	Abbreviated IES-R (IES-R 6)	Moderate-severe symptoms, >10; this is the cutoff assumed for PTSD diagnosis	Point prevalence: 708 (36.0)
Brady et al,^22^ 2022	Ireland	CSS	181	6	IES-R	Moderate to severe symptoms: ≥26; assumed to be cutoff relevant for PTSD diagnosis (not explicitly mentioned)	Point prevalence: 29 (16.0)
Pazmino Erazo et al,^23^ 2021	Ecuador	CSS	557	NR	IES-R	Mild, 9-25; moderate, 26-43; severe, >44; cutoff considered moderate score	Point prevalence: mild: 173 (31.1); moderate: 139 (25.0); severe: 111 (19.9)
Oz Tunçer et al,^24^ 2022	Turkey	CSS	232	57.3	IES-R	Mild, 24-32; moderate, 33-37; severe, >37; best value for cutoff, ≥33	Point prevalence: mild, 40 (18); moderate: 33 (14.2); severe: 122 (52.6)
Holzer et al,^25^ 2021	US	CSS	222	16.2	IES-R	Clinically meaningful PTSD symptoms, ≥24	Point prevalence: 20 (9.0)
Meena et al,^26^ 2022	India	CSS	39	NR	IES-R	Clinical relevance for PTSD, >24	Point prevalence: 1 (2.6)
Gilleen et al,^27^ 2021	UK	CSS	386	19.8	IES-R	High PTSD symptoms, ≥26; high PTSD symptoms assumed to be indicative of PTSD diagnosis, thus cutoff	Point prevalence: 64 (16.6)
Chatzittofis et al,^28^ 2021	Cyprus	CSS	178	NR	IES-R	Clinically relevant PTSD symptoms, >33	Point prevalence: 13 (7.3)
Civantos et al,^29^ 2020	US	CSS	349	10.22 for residents and 6.46 for attendings	IES-R	Mild, 9-25; moderate, 26-43; severe, 44-75; PTSD cutoff assumed to be considered the moderate score	Point prevalence: mild, 114 (32.7); moderate, 73 (20.9); severe, 23 (6.6)
Ahmed et al,^30^ 2022	Pakistan	CSS	105	NR	IES-R	Of clinical concern, NA; mild to moderate PTSD, NA; severe PTSD, NA; there was no cutoff score provided	Point prevalence: of clinical concern, 16 (15.2); mild to moderate PTSD, 10 (9.5); severe PTSD, 19 (18.1)
Pasin et al,^31^ 2020	Italy	CSS	503	38.8	IES-R	Probable diagnosis of PTSD, ≥33	Point prevalence: 107 (21.3)
Das et al,^32^ 2021	India	CSS	303	NR	IES-R	Clinical concern for PTSD, 24-32; probable PTSD, 33-36; definitive PTSD, >37; cutoff considered ≥33	Point prevalence: clinical concern for PTSD, 50 (16.5); probable PTSD, 26 (8.6); definitive PTSD: 107 (35.3)
Costantini et al,^33^ 2021	Italy	CSS	101	NR	IES-R	Probable diagnosis of PTSD, ≥33	Point prevalence: 33 (32.7)
Asnakew et al,^34^ 2021	Northwest Ethiopia	CSS	77	93.6	IES-R	PTSD as a clinical concern, ≥24; clinical concern PTSD symptoms assumed to be indicative of PTSD diagnosis	Point prevalence: 21 (27.3)
Gorini et al,^35^ 2022	Italy	CSS	265	NR	IES-R	Mild, 24-32; moderate, 33-36; severe, 37-88; moderate score considered cutoff (≥33)	Point prevalence: mild: 49 (18.5); moderate: 14 (5.3); severe: 60 (22.6)
Roberts et al,^36^ 2021	UK and Ireland	Prospective Longitudinal cohort study	5440	Peak response rate, 71.6; deceleration response rate, 56.6	IES-R	Probable PTSD, >33	Point prevalence: ≥24 (peak), 647 (23.7); ≥24 (deceleration), 484 (17.l7); ≥33 (peak), 343 (12.6); ≥33 (deceleration), 276 (10.1)
Tan et al,^37^ 2021	Global (101 countries)	CSS	3391	79.2	IES-R	Mild, mean score of 2 from each subscale; moderate, mean score of 3 from each subscale; severe, mean score of 4 from each subscale; implied cutoff: ≥44	Point prevalence: 753 (22.2)
Lasalvia et al,^38^ 2021	Verona, Italy	Longitudinal	April-May 2020: 667; April-May 2021: 309	April-May 2020: 36.9; April-May 2021: 17.4	IES-R	Clinical PTSD, ≥24	April to May 2020; 141 (21.1); April to May 2021, 66 (21.4)
Azoulay et al,^39^ 2021	France	CSS	272	70.2	IES-R	Symptoms of PTSD, ≥26; PTSD symptoms assumed to be indicative of PTSD diagnosis, thus cutoff ≥26	Point prevalence: 64 (23.5)
Mehta et al,^40^ 2022	Canada	CSS	63	NR	IES-R	Probable PTSD, ≥33	Point prevalence: 3 (4.8)
Li et al,^41^ 2022	China	CSS	37	NR	PCL-C	Possible PTSD, ≥44	Point prevalence: 4 (10.8)
Gainer et al,^42^ 2021	US	CSS	1724	NR	APCL	Clinically meaningful threshold for PTSD, ≥14	Point prevalence: 474 (27.5)
Chang et al,^43^ 2021	US	Longitudinal cohort study^a^	31	50	PCL-5	Severe enough condition to benefit from PTSD treatment, ≥ 31	Point prevalence: 11 (35.5)
Martsenkovskyi et al,^44^ 2022	Ukraine	CSS	281	46.8	PCL-5	Meets criteria for PTSD, ≥31	Point prevalence: 59 (21.0)
Hendrickson et al,^45^ 2022	US	CSS	60	NR	PCL-5	Clinical PTSD range, ≥31	Point prevalence: 11 (20.8); however, this included the 53 who had data
Martinez-Caballero et al,^46^ 2021	Spain	CSS	61	37.6	DTS-8	Suspicion of PTSD, ≥12	Point prevalence: 15 (24.6)
She et al,^47^ 2022	China	CSS	423	60	PSS-SR	Likelihood of posttraumatic stress symptoms, ≥13	Point prevalence: 189 (44.7)
Gonzalez-Mesa et al,^48^ 2021	Spain	CSS	220	27.5	ITQ	To reach a diagnosis of PTSD, at least 1 of 2 symptoms from each PTSD symptom subscale (score ≥2), and at least 1 of the functional impairment items	Point prevalence: 27 (12.3)
Baumann et al,^49^ 2021	US	Longitudinal cohort study	First survey, 426; follow-up survey: 262	First survey, 56.7; follow-up survey, 61.5	PC-PTSD	Increased risk for PTSD, ≥3	First survey, 85 (32.8); follow-up survey: 66 (25.9)
Villalba-Arias et al,^50^ 2021	Paraguay	CSS	218	48.8	PCL-C	Diagnostic cutoff, ≥50	Point prevalence: 10 (4.6)
Greenberg et al,^51^ 2021	UK	CSS	291	NR	PCL-6	Probable PTSD, >13	Point prevalence: 92 (31.6)
Leon Rojas et al,^52^ 2022	Mexico	CSS	303	NR	PCL-5	Provisional PTSD, ≥33 or by treating each item rated as 2 or higher as an endorsed symptom, then following the DSM-5 diagnostic rules	59 (19.5)
Kalyanaraman et al,^53^ 2021	US	CSS	294	16.0	PCL-5	Positive PTSD, ≥31	Point prevalence: 23 (7.8)
Marco et al,^54^ 2020	US	CSS	1154	4.7	PCL-5	Symptom suggestive of PTSD, ≥33	Point prevalence: 257 (22.3)
Bahadirli et al,^55^ 2021	Turkey	CSS	406	16.7	PCL-5	Probable diagnosis of PTSD, ≥47	Point prevalence: 93 (22.9)
Lombard et al,^56^ 2022	South Africa	CSS	391	23.8	PCL-5	Provisional diagnosis of PTSD, ≥33	Point prevalence: 69 (17.6)
Guo et al,^57^ 2021	China	CSS	202	63.8	PCL-C	Mild, 38-49; severe, 50-85; cutoff (as stated by authors), ≥37	Point prevalence: mild, 20 (9.9); severe, 7 (3.5)
Yang et al,^58^ 2022	China	CSS	396	88.2	PCL-5	Possibility of PTSD, ≥31	Point prevalence: 42 (10.6)
Kaplan et al,^59^ 2021	New York City, US	CSS	560	56.6	PCL4-5	Positive PTSD screen, ≥8	Point prevalence: 74 (13.2)
Bates et al,^60^ 2021	UK	CSS	24	60.3	PCL-5	Provisional diagnosis of PTSD, ≥31	Point prevalence: 2 (8.3)
Huang et al,^61^ 2021	China	Comparative study of scales but refernce standard was used	136	96.2	PCL-5	Probable PTSD diagnosis, ≥33	Point prevalence: 57 (41.9)
Dehon et al,^62^ 2021	US	CSS	255	50	PCL-5	Probable PTSD diagnosis: ≥31	Point prevalence: 19 (7.5)
Stafseth et al,^63^ 2022	Norway	CSS	43	NR	PCL-5	Probable PTSD diagnosis, ≥31	Point prevalence: 1 (2.3)
Machado et al,^64^ 2022	Brazil	CSS	372	67.3	PCL-5	Probable PTSD, ≥36	Point prevalence: 77 (20.7)
Ouazzani et al,^65^ 2021	Morocco	CSS	1267	63.3	PCL-5	Probable PTSD, ≥33	Point prevalence: 276 (21.8)
Piacentini et al,^66^ 2022	Italy	CSS	895	NR	PCL-5	A provisional PTSD diagnosis can be made by treating each item rated as 2 meaning moderately or higher as a symptom endorsed, then following the *DSM-5* diagnostic rule, which requires at least 1 B item (questions 1-5), 1 C item (questions 6-7), 2 D items (questions 8-14), 2 E items (questions 15-20); thus, cutoff implied to be ≥40	Point prevalence: 100 (11.2)
Seifeldin et al,^67^ 2022	Egypt	CSS	124	NR	PCL-C	Mild PTSD symptoms, 28-29; moderate to moderately high severity of PTSD symptoms, 30-44; high severity of PTSD symptoms, ≥45; cutoff for PTSD case, ≥47	Point prevalence: 47 (37.9)
Guo et al,^68^ 2022	China	CSS	427	NR	PC-PTSD	Probable PTSD, ≥2	Point prevalence: 89 (20.8)
Mosheva et al,^69^ 2021	Central Israel	CSS	349	NR	PC-PTSD-5	Probable PTSD, ≥3	Point prevalence: 33 (9.5)
Schwartz et al,^70^ 2022	New York, United States	CSS	596	4.9	PC-PTSD-5	Probable PTSD, ≥3	Point prevalence: 135 (22.7)
Isik et al,^71^ 2021	Turkey	CSS	352	NR	PTSD-SS	Clinical significance for PTSD, ≥24	Point prevalence: 13 (3.7)
Kader et al,^72^ 2021	Qatar	CSS	7	86.7	PDS-5	Probable diagnosis for PTSD, ≥28	Point prevalence: 2 (28.6)
Real-Ramirez et al,^73^ 2020	Mexico	CSS	175	NR	TOP-8	Mild risk, 5-17; moderate risk, 18-25; severe risk with probability of comorbidities: 26-36; moderate risk considered cutoff (ie, ≥18)	Point prevalence: mild risk, 105 (60.0); moderate risk, 19 (10.9); severe risk with probability of comorbidities, 3 (1.7)

Abbreviations: APCL, abbreviated PCL; CSS, cross-sectional study; DTS-8, Davidson Trauma Scale; DSM-5, *Diagnostic and Statistical Manual of Mental Disorders* (Fifth Edition); IES-R, Impact of Event Scale-Revised; NA, not applicable; NR, not reported; PC-PTSD, primary care PTSD; PCL, PTSD Checklist; PCL4-5, 4-item PCL for the *DSM-5*; PCL-5, PCL for *DSM-5*; PCL-C, PCL-civilian; PDS-5, Posttraumatic Diagnostic Scale for *DSM-5*; PSS-SR, PTSD Symptom Scale Self-Report Version; PTSD, posttraumatic stress disorder; PTSD-SS, PTSD Short Scale; TOP-8, 8-item Treatment Outcome PTSD Scale.

^a^ Longitudinal data not applicable since pre-COVID-19 pandemic.

### Data Selection and Extraction, Extraction, Synthesis, and Risk of Bias Assessment

Detailed methods regarding data selection, extraction, and synthesis are presented in eAppendix 2 in Supplement 1. Risk of bias was assessed using a modified Newcastle Ottawa Scale (NOS) criteria based on Mata et al.^16^

### Statistical Analysis

The prevalence of PTSD from 57 studies was pooled and analyzed for heterogeneity. For meta-analyses, the results of each study were treated as dichotomous variables with any individual with PTSD or PTSD symptoms above a clinical threshold considered as a positive screen. Based on the large degree of heterogeneity, a Baujat plot was conducted to see if the omission of any studies would mitigate any heterogeneity. A random effects model was subsequently used to account for the heterogeneity when constructing forest plots and odds ratios (OR). Pooled results were calculated for all included studies and subgroups of sex, specialty, and career stage via a random intercept logistic regression model. To calculate pooled OR estimates, the Mantel-Haenszel method was used to determine the weight of each study and Knapp-Hartung adjustments were made for the random effects model. The average prevalence across the surveys was used for prospective cohort and longitudinal studies that reported proportional estimates throughout the course of the study. Prevalence of each study was stratified by year(s) of data collection to understand how the development of PTSD symptoms changed throughout the COVID-19 pandemic. Heterogeneity was assessed by the *I^2^* statistic. Results were presented as forest plots. All analyses were conducted using R version 4.2.2 (R Foundation for Statistical Computing) with RStudio. All statistical tests were 2-sided at the α < .05. Sensitivity analyses were done to account for sample size, response rate, PTSD scale, risk of bias, and geographical location (continent).

## Results

### Study Characteristics

Our search of MEDLINE, Embase, and PsycInfo yielded 24 629 records. After screening titles and abstracts, 357 were deemed eligible for full-text review. Of these, 39 studies were unable to be retrieved, 3 were duplicates, 75 lacked a clear outcome definition (eg, a validated questionnaire was not used) or had the wrong outcome, 111 were not original articles (eg, comments, letters, and reviews), 2 were not in English, and 70 included mixed populations (eg, health care workers without separate data reported for physicians). In total, 57 studies satisfied the inclusion and exclusion criteria of this study (Table 1), involving a total of 28 965 physicians.^17,18,19,20,21,22,23,24,25,26,27,28,29,30,31,32,33,34,35,36,37,38,39,40,41,42,43,44,45,46,47,48,49,50,51,52,53,54,55,56,57,58,59,60,61,62,63,64,65,66,67,68,69,70,71,72,73^ Fifty-five studies (96.4%)^17,18,19,20,21,22,23,24,25,26,27,28,29,30,31,32,33,34,35,36,37,39,40,41,42,43,44,45,46,47,48,50,51,52,53,54,55,56,57,58,59,60,61,62,63,64,65,66,67,68,69,70,71,72,73^ were cross-sectional while 2 (3.5%) were longitudinal.^38,49^ Thirty-three (57.8%) studies provided a response rate with a mean of 47.5% (median, 50%; range, 4.7%-96.2%).^7,22,24,25,27,29,31,34,36,37,38,39,43,44,46,47,48,49,50,53,54,55,56,57,58,59,60,61,62,64,65,70,72^

The number of participants in each study ranged from 7 to 5440 (mean, 508; median, 291). Seventeen studies were in Europe (UK, 5; Italy, 5; Spain, 2; Ireland, 2; France, 1 in each: Norway, Ukraine, Cyprus),^20,22,27,28,31,33,35,36,38,39,44,46,48,51,60,63,66^ 16 in Asia (China, 7; India, 3; Turkey, 3; 1 in each: Pakistan, Israel, Qatar),^17,19,24,26,30,32,41,47,55,57,58,61,68,69,71,72^ 4 in Africa (1 in each: Ethiopia, South Africa, Egypt, Morocco),^34,56,65,67^ 2 in Australia,^18,21^ 14 in North America (US, 11; Mexico, 2; Canada, 1),^25,29,40,42,43,45,49,52,53,54,59,62,70,73^ 3 in South America (1 in each: Brazil, Paraguay, Ecuador).^23,50,64^ One study by Tan et al^37^ was a global study with respondents from 101 countries. Additionally, 43 studies (75.4%) did not specify medical specialties,^17,18,19,20,21,22,23,24,25,26,27,28,30,32,33,34,35,38,39,40,41,44,45,46,47,48,50,51,52,53,55,57,58,60,61,63,64,67,69,70,71,72,73^ 8 studies (14.0%)^29,43,49,54,56,62,66,68^ focused on 1 specialty, and 6 studies (10.5%) reported on physicians of various specialties.^31,36,37,42,59,65^

All studies identified PTSD via self-report from validated questionnaires: 24 studies (42.1%) by the Impact Events Scale-Revised (IES-R),^17,18,19,20,21,22,23,24,25,26,27,28,29,30,31,32,33,34,35,36,37,38,39,40^ 23 studies (40.3%) by the PCL (PCL-5, 16 studies; PCL-civilian [PCL-C], 4 studies; PCL-[abbreviated 6-item], 1 study; Abbreviated PCL, 1 study; 4-item PCL-5, 1 study),^41,42,43,44,45,50,51,52,53,54,55,56,57,58,59,60,61,62,63,64,65,66,67^ 4 studies (7%) by primary care (PC)-PTSD (PC-PTSD, 2 studies; PC-PTSD-5, 2 studies),^49,68,69,70^ and 6 studies (11%) by 1 of the following: Davidson Trauma Scale, Posttraumatic Diagnostic Scale for *DSM-5*, PTSD Symptom Scale Self-Report version, PTSD Short Scale, 8-item Treatment Outcome PTSD Scale, and International Trauma Questionnaire.^46,47,48,71,72,73^ No studies used more than 1 questionnaire. The definition of PTSD varied across studies or scales used with the full scoring criteria are presented in Table 1. For IES-R, 11 studies classified PTSD as scores 33 or more or more than 33,^18,19,20,24,28,31,32,33,35,36,40^ 4 studies as 24 or more or more than 24,^25,26,34,38^ 5 studies as 26 or more,^22,23,27,29,39^ 2 studies as 44 or more,^17,37^ 1 study study used an abbreviated IES-R scale and had a cutoff of more than 10,^21^ and 1 study did not report a cutoff scale.^30^ For the latter, cutoffs were implied based on the scale cited or using the score for moderate symptoms. This approach was applied to all scales when cutoffs were not clearly reported. The IES-R has previously reported a sensitivity of 80% and a specificity of 72% for a low cutoff score of 23.^74^ All reported cutoff scores in this review were above 23, and higher cutoff values display greater sensitivity and specificity^75^; thus, the studies included have appreciable diagnostic accuracy for PTSD. For PCL-5, 8 studies defined PTSD with scores as 31 or more,^43,44,45,53,58,60,62,63^ 5 studies as 33 or more,^52,54,56,61,65^ 1 study as 47 or more,^55^ 1 study as 40 or more,^66^ and 1 study as 36 or more.^64^ Scores of 23 have shown to have strong sensitivity and specificity (0.82 and 0.70, respectively),^76^ with higher scores associated with higher sensitivity and specificity.^77^ Additionally, 1 study used the abbreviated PCL-5 scale with a cutoff of 8,^59^ and 1 study used the abbreviated PCL-5 scale with a cutoff of 14.^42^ Furthermore, for the 4 PCL-C studies, 1 study used a cutoff of 44,^41^ 1 study used cutoff of 47,^67^ 1 study used a cutoff of 50 or more,^50^ and 1 study used a cutoff of 37 or more.^57^ A cutoff score of 26 displays respectable specificity of 0.63 and sensitivity of 0.86, with higher scores having greater specificity rates.^78^ Both studies that used PC-PTSD-5 had a cutoff score of 3 or more, which has a respectable sensitivity and specificity of 1.00 and 0.69, respectively.^79^ Furthermore, the 2 studies that used PC-PTSD had cutoff scores or 2 or more (sensitivity of 0.94 and specificity of 0.69) or 3 or more (sensitivity of 0.85 and specificity of 0.82).^80^ The cutoffs for the other scales used also had appropriate diagnostic accuracy. For instance, 1 study^46^ used the Posttraumatic Diagnostic Scale (abbreviated 8-item) with a cutoff of 12, which has a sensitivity of 0.77 and specificity of 0.70.^81^

### Extent of PTSD in Physicians During the COVID-19 Pandemic

**Figure 2.**
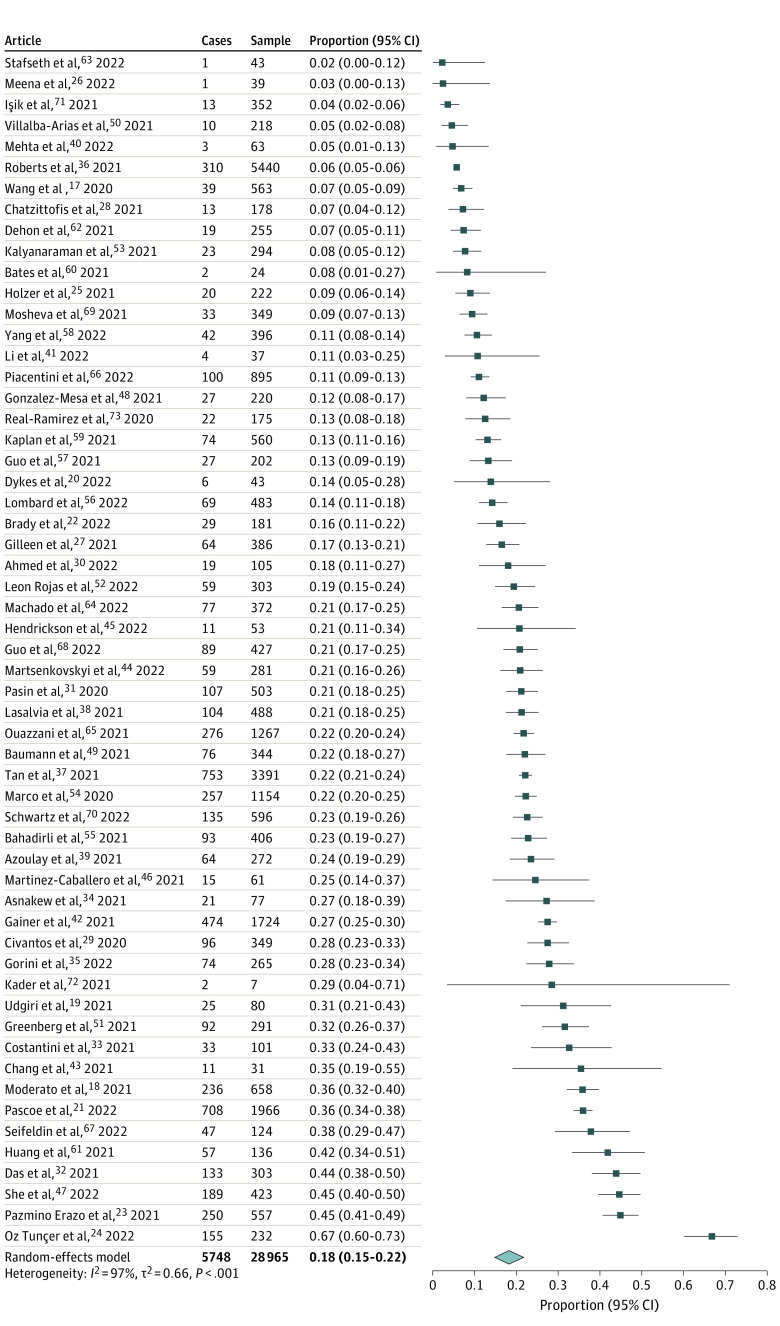
Prevalence of PTSD Among Physicians During the COVID-19 Pandemic Across All Studies PTSD indicates posttraumatic stress disorder.

**Figure 3.**
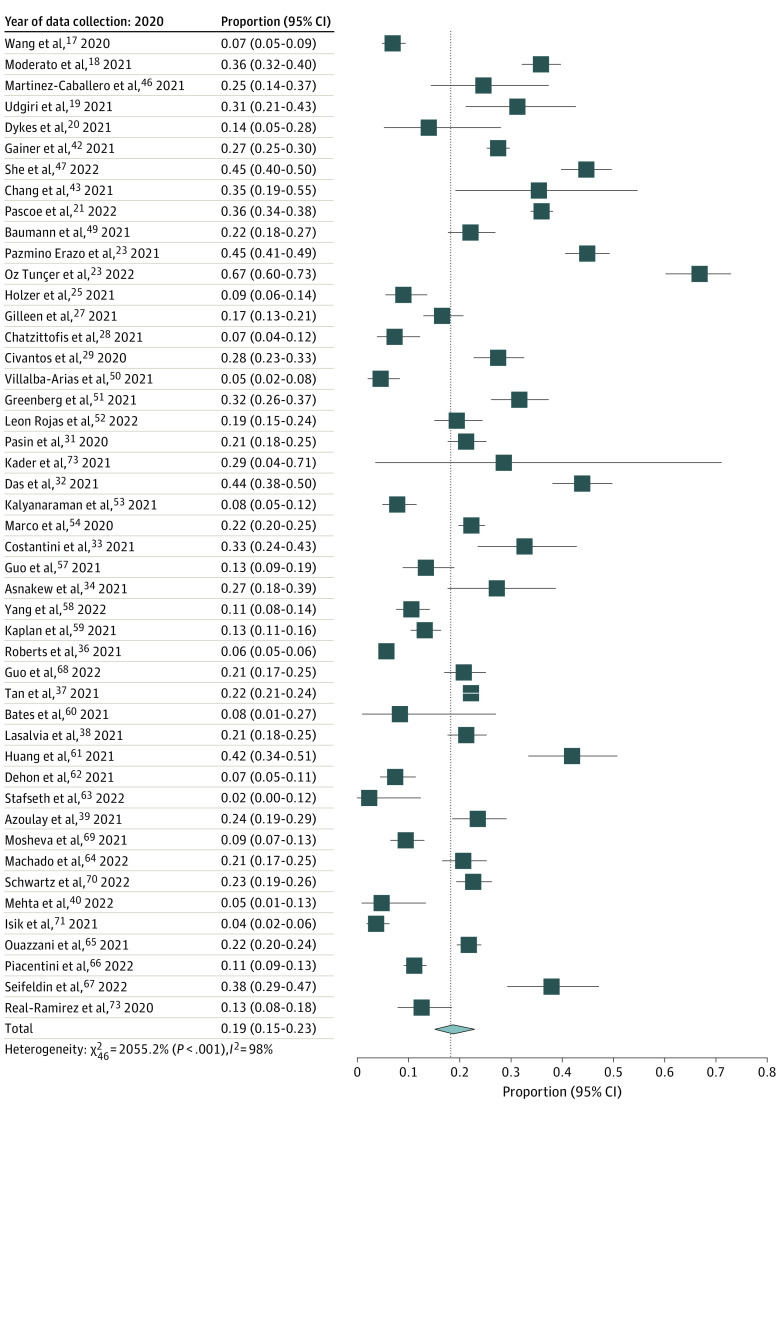
Prevalence of PTSD Across Different Studies Stratified by Their Data Collection Periods PTSD indicates posttraumatic stress disorder.

For the 28 965 physicians in the included studies, 5748 (pooled prevalence, 18.3%; 95% CI, 15.2%-21.8%) were identified to have probable or clinically concerning PTSD with high heterogeneity of prevalence across studies (τ = 0.811; τ^2^ = 0.657; *I^2^* = 97.3%; Q Wald-type, 2107.4) (Figure 2). The proportion of cases ranged from 3% to 67% for the IES-R scale, 2.3% to 41.9% for PCL, and 9.5% to 22.7% for PC-PTSD. All studies had different data collection periods throughout the COVID-19 pandemic. Most studies were conducted early in the pandemic (46 [80.7%]) during 2020,^17,18,19,20,21,23,24,25,27,28,29,31,32,33,34,36,37,39,40,42,43,46,47,48,49,50,51,52,53,54,57,58,59,60,61,62,63,64,65,66,67,69,70,71,72,73^ while 6 studies (10.5%) were conducted in 2021,^22,26,35,38,44,68^ 4 studies (7.0%) during 2020 and 2021,^30,45,55,56^ and 1 study during 2021 and 2022.^41^ The overall prevalence did not statistically differ between different periods (Figure 3). Sensitivity analyses demonstrated similar results when stratifying by sample size of more than 200 participants (18.7%; 95% CI, 15.1%-23.1%) (eFigure 1 in Supplement 1) and a response rate of at least 50.0% (19.9%; 95% CI, 13.9%-27.7%) (eFigure 2 in Supplement 1). Sensitivity analyses were also conducted by continent and PTSD scale used (eFigures 3 and 4 in Supplement 1).

### Differences by Sex

Fourteen studies (22.8%) stratified PTSD outcomes by sex (Table 2),^18,19,29,31,37,42,48,49,54,56,59,65,67,70^ with a pooled total of 3540 male and 4308 female participants. Among the 14 studies, 12 (92.3%) reported a higher prevalence in females compared with males (difference: mean, 12.0%; median, 13.9%; range, 1.9% to 23.5%),^18,19,29,31,37,42,48,49,54,56,67,70^ 1 study (7.1%) reported the same prevalence across both sexes,^59^ and 1 study (7.1%) reported that male physicians had higher prevalence of PTSD than female physicians, by 0.09%.^65^ The overall prevalence for females included a mean of 26.9%, a median of 30.8%, and a range of 2.5% to 46.3%. The overall prevalence for males included a mean of 18.8%, a median of 19.2%, and a range of 5.7% to 29.7%. Moreover, the pooled OR for female (vs male) physicians developing PTSD was 1.93 (95% CI, 1.56-2.39; *P* < .001) (eFigure 6 in Supplement 1).

**Table 2.** Studies Assessing PTSD in Physicians by Age and Sex

Source	Location	M, No. (%)	F, No. (%)	Outcome assessment	Definition of outcome	Outcome	Age distribution, No. (%)	Outcome, No. (%)
Moderato et al,^18^ 2021	Australia	108 (16.4)	550 (83.6)	IES-R	Clinical PTSD cutoff: ≥3	M, 24 (22.2); F, 212 (38.5)	<30 y: 51 (7.8); 30-40 y: 294 (44.7); >40 y: 313 (47.6)	<30: 13 (25.5) 30-40: 124 (42.2) >40: 99 (31.6)
Udgiri et al,^19^ 2021	India	37 (46.3)	43 (53.8)	IES-R	Mild, ≥24; moderate ≥33; severe ≥37; moderate considered the best cutoff for probable PTSD diagnosis	Mild: M, 23 (62.2); F, 19 (44.2); moderate: M, 6 (16.2); F, 10 (23.3); severe: M, 5 (13.5); F, 4 (9.3)	NA	NA
Civantos et al,^29^ 2020	US	212 (60.7)	137 (39.3)	IES-R	Mild, 9-25; moderate, 26-43; severe, 44-75; PTSD cutoff assumed to be considered the moderate score	Mild: M, 67 (31.6); F, 39 (28.5); moderate: M, 32 (15.1); F, 41 (29.9); severe: M, 13 (6.1); F, 10 (7.3)	26-30 y, 94 (26.9); 31-35 y, 114 (32.7); 36-40 y, 44 (12.6); >40 y, 97 (27.8)	26-30 y: Mild, 26 (27.7); moderate, 22 (23.4); severe, 8 (8.5); 31-35 y: mild, 36 (31.6); moderate, 26 (22.8); severe, 3 (2.6); 36-40 y: mild, 13 (29.5); moderate, 8 (18.2); severe, 4 (9.1); >40 y: mild, 39 (40.2); moderate, 17 (17.5); severe, 8 (8.2)
Pasin et al,^31^ 2020	Italy	217 (43.2)	285 (56.8)	IES-R	Probable diagnosis of PTSD, ≥33	M, 29 (13.4); F, 78 (27.3)	<30 y, 239 (47.6); 30-35 y, 235 (46.8); 35-45 y, 27 (5.4); 45-55 y, 0; >55 y 2 (0.4)	<30 y, 41 (17.2); 30-35 y, 60 (25.5); 35-45 y, 6 (22.2); 45-55 y, 0; >55 y: 0
Marco et al,^54^ 2020	US	679 (59.5)	462 (40.5)	PCL-5	Symptoms suggestive of PTSD, ≥33	M, 146 (21.5); F, 108 (23.4)	<30 y, 30 (2.6); 30-39 y, 323 (28.0); 40-49 y, 317 (27.6); 50-59 y, 269 (23.3); ≥60 y, 199 (17.2)	<30 y, 6 (20.0); 30-39 y, 79 (24.5); 40-49 y, 81 (25.6); 50-59 y, 62 (23.0); ≥60 y, 24 (12.1)
Lombard et al,^56^ 2022	South Africa	199 (50.9)	192 (49.1)	PCL-5	Provisional diagnosis of PTSD, ≥33	M, 22 (11.1); F, 46 (24.2)	<30 y, 9 (2.3); 30-39 y, 139 (35.5); 40-49 y, 106 (27.1); ≥50 y, 137 (35.0)	<30 y, 1 (11.1); 30-39 y, 36 (25.9); 40-49 y, 17 (16.0); ≥50 y, 15 (10.9)
Kaplan et al,^59^ 2021	US	278 (49.8)	280 (50.2)	PCL4-5	Positive screening of PTSD, ≥8	M, 36 (12.9); F, 36 (12.8)	<35 y, 512 (91.8); ≥35 y, 48 (8.2)	<35 y, 66 (12.9); ≥35 y, 8 (16.7)
Ouazzani et al,^65^ 2021	Morocco	516 (40.7)	752 (59.3)	PCL-5	Probable PTSD, ≥33	M, 83 (16.1); F, 193 (25.7)	≤30 y, 788 (62.1); >30 y, 478 (37.7)	≤30 y, 165 (20.9); >30 y, 111 (23.2)
Seifeldin et al,^67^ 2022	Egypt	44 (35.5)	80 (64.2)	PCL-C	Cutoff for PTSD case, ≥47	M, 10 (22.7); F, 37 (46.3)	<34 y, 77 (62.1); >35 y, 47 (37.9)	<34, 29 (37.7); >35 y, 18 (38.3)
Leon Rojas et al,^52^ 2022	Mexico	0	303 (100)	PCL-5	Provisional PTSD; ≥33 or by treating each item rated as 2 or higher as an endorsed symptom, then following the *DSM-5* diagnostic rules	F, 59 (19.5)	NA	NA
Gainer et al,^42^ 2021	US	750 (43.9)	958 (56.1)	APCL	Clinically meaningful threshold for PTSD, ≥14	M, 141 (18.8); F, 331 (34.5)	26-30 y: 217 (12.7); 31-40 y, 614 (35.8); 41-50 y, 402 (23.5); 51-60 y, 260 (15.2); >60 y, 221 (12.9)	26-30 y, 67 (31.1); 31-40 y, 194 (31.6); 41-50 y, 114 (28.3); 51-60 y, 60 (23.2); >60 y, 36 (16.3)
Baumann et al,^49^ 2021	US	First survey, 129 (50.4); follow-up, 130 (50.2)	127 (50.0)	PC-PTSD	Increased risk for PTSD, ≥3	Initial survey: M, 29 (22.5); F, 55 (43.3); follow-up survey: M, 21 (16.8); F, 44 (34.7)	NA	NA
Schwartz et al,^70^ 2022	US	303 (50.8)	293 (49.2)	PC-PTSD-5	Probable PTSD, ≥3	M, 48 (15.8); F, 87 (29.7)	NA	NA
Gonzalez-Mesa et al,^48^ 2021	Spain	70 (31.9)	149 (68.0)	ITQ	PTSD diagnosis ≥2 in each subscale as well as functional impairment	M, 4 (6.0); F, 23 (15.3)	≤55 y, 146 (66.7); >55 y, 71 (32.4)	≤55 y, said to be significantly less prevalent than those >55 y; >55 y, 15 (21.1)

Abbreviations: *DSM-5*, *Diagnostic and Statistical Manual of Mental Disorders* (Fifth Edition); F, female; IES-R, Impact of Event Scale-Revised; ITQ, International Trauma Questionnaire; M, male; NA, not applicable; PC-PTSD, primary care; PCL4-5, 4-item PCL-5; PCL-5, PTSD Checklist for *DSM-5*; PCL-6, PCL-(abbreviated 6-item); PCL-C, PCL-civilian; PSS-SR, PTSD Symptom Scale Self-Report version; PTSD, posttraumatic stress disorder.

### Differences by Age

Ten studies (17.5%) stratified PTSD by age.^18,29,31,42,48,54,56,59,65,67^ Eight of the studies (80.0%) reported a lower prevalence of PTSD among young physicians compared with older age groups (Table 2).^18,31,48,54,56,59,65,67^ Among these 8 studies, 4 (50.0%) of them showed that the prevalence of PTSD was highest in those aged 30 years or older,^18,31,56,65^ while the other 4 studies (50.0%) showed the prevalence was highest in the following age groups: younger than 50 years,^54^ 35 years or older,^59^ older than 35 years,^67^ and older than 55 years.^48^ In contrast, 2 studies showed that their lowest age group (both 30 years or younger) had a higher prevalence of PTSD.^29,42^ Of note, the absolute difference in the proportions of PTSD between the youngest and older groups ranged from 0.6% to 11.2% (mean, 4.8%) for all 10 studies. Due to the large heterogeneity between studies, a meta-analysis was not conducted for this factor.

### Physicians in Training vs Attending

Sixteen studies (28.1%)^19,21,23,29,31,32,36,37,38,39,42,54,56,59,65,66^ stratified PTSD by career stage (eTable 1 in Supplement 1), with a pooled total of 4148 medical trainee and 7038 attending physician participants. Twelve studies (75.0%) stratified by attending vs doctors in training (ie, interns, residents, and fellows),^21,23,29,32,36,37,38,39,42,56,65,66^ 2 (12.5%) by trainees or residents,^19,31^ and 2 (12.5%) by level of physicians.^54,59^ Prevalence of PTSD among trainees ranged from 19.2% to 46.0% (mean, 31.0%) and attendings ranged from 12.2% to 42.5% (mean, 24.7%). Trainees, compared with attendings, had an OR of 1.33 (95% CI, 1.12-1.57) residual heterogeneity (57.7%) of having PTSD (eFigure 7 in Supplement 1).

### Specialty

Thirteen studies (22.8%) stratified PTSD by 1 or more specialties (eTable 1 in Supplement 1).^29,31,36,42,43,49,54,56,59,62,65,66,68^ The most common specialties were emergency medicine (7 studies [12.3%])^31,36,42,43,49,54,62^ and anesthesiology (6 studies [10.5%]).^31,36,42,56,66,68^ Internal medicine, otolaryngology, family medicine, surgery, and critical care were reported in multiple studies whereas certain specialties (eg, neurology) were reported in only 1 study.^42^ More studies focused on specialties with a greater likelihood of treating patients with COVID-19, such as emergency medicine and anesthesiology. The mean (range) of PTSD prevalence for each specialty was reported as follows: emergency medicine, 23.4% (5.4%-35.5%); anesthesiology, 16.1% (2.6%-21.8%); surgery, 22.4% (9.8%-35.0%); family medicine, 31.2% (31.2%-31.3%); internal medicine, 21.9% (16.7%-27.9%); critical care, 14.8% (2.4%-27.1%); and otolaryngology, 23.0% (18.5%-27.5%) (eFigure 8 in the Supplement 1). Three studies that reported results stratified by the front line specialties of emergency medicine, anesthesiology, and critical care found that emergency medicine had the highest proportion of PTSD.^31,42,59^

### Risk Of Bias Assessment

A modified NOS was used for assessing the quality of cohort and cross-sectional studies (eTable 2 in the Supplement 1).^16^ Eight studies (14.0%) were marked as having a high risk of bias,^19,20,26,30,33,43,60,72^ while 49 were marked as having low risk of bias.^17,18,21,22,23,24,25,27,28,31,32,34,35,36,37,38,39,40,41,42,44,45,46,47,48,49,50,51,52,53,54,55,56,57,58,59,61,62,63,64,65,66,67,68,69,70,71,72,73^ Issues of bias were mostly due to low response rate or sample size, recruitment from only 1 site, and a lack of comparability of nonrespondents. A sensitivity analysis was conducted on low risk of bias studies (49 studies [86.0%]) and also found a PTSD prevalence of 18.2% (95% CI, 14.9%-22.0%) (eFigure 5 in Supplement 1).^17,18,21,22,23,24,25,27,28,31,32,34,35,36,37,38,39,40,41,42,44,45,46,47,48,49,50,51,52,53,54,55,56,57,58,59,61,62,63,64,65,66,67,68,69,70,71,72,73^

## Discussion

The COVID-19 global pandemic was the largest modern-day pandemic and provided a unique opportunity to study mental health impacts on health care workers. In this systematic review and meta-analysis of physicians during the COVID-19 pandemic, we found PTSD to be more than 3 times higher than the general population and higher than the historically reported PTSD for physicians before the COVID-19 pandemic.^1,2^ Studies were survey-based and highly variable because of the different scales used, different cutoffs among those scales, and the severity of regions affected by the pandemic.

Our findings expand and update on work conducted by Qi et al^82^ who reported a point-prevalence of 31% (95% CI, 21%-40%) for physicians with PTSD. While this is greater than what was found in this study, the difference between these findings may be attributed to Qi et al^82^ reporting earlier in the pandemic when disease severity was higher. The current study expanded on previous research by including more studies, more contemporary publications, broader geographic representation, and details on relevant physician-related characteristics.

Assessing the prevalence of PTSD among physicians is challenging. Wide variation in the method used to identify PTSD (multiple different assessment scales with different definitions for a positive screen) preclude a definitive estimate of the true prevalence of PTSD after the COVID-19 pandemic. For instance, almost all studies used either a variation of the IES-R (24 studies) or PCL scales (23 studies), but the convergent validity of the 2 scales were weakly moderate (*r* = 0.58).^83^ Furthermore, the cutoffs between studies that used the same scales greatly differed, such as for the IES-R. Asnakew et al,^34^ Lombard et al,^56^ and Bahadirli et al^55^ used cutoffs of 24 or more, 33, and 47, respectively. Consensus on the cutoffs for PTSD identification in self-report questionnaires for PTSD would allow for a more accurate assessment of the prevalence of PTSD in physicians. Additional population-based cohort studies are required to determine the true prevalence of PTSD within physician populations during the COVID-19 pandemic. Such research can be highly advantageous in informing health care systems wellness support for physicians during future pandemics, as well as general wellness programs to combat burnout and other mental health issues prevalent in physicians.

Female physicians were significantly more likely to develop PTSD than men (OR, 1.93; 95% CI, 1.56-2.39). This is consistent with the general public,^84^ where females are approximately 2 times more likely than males to develop PTSD.^5,84^ Possibilities proposed in the general population include a higher reliance on social support to manage stress, which was likely significantly limited during the COVID-19 pandemic,^85^ and a stronger caregiver role with associated strain.^48^ Additional research is needed to understand why female physicians experience more PTSD than male physicians and assess whether female physicians have an added risk of PTSD compared with females who are not physicians.

Medical trainees were significantly more susceptible to developing PTSD (OR, 1.33; 95% CI, 1.12-1.57). This finding aligns with similar studies conducted prior to the COVID-19 pandemic, such as Jackson et al,^86^ who reported higher rates of PTSD among surgical residents relative to attendings. A plausible mechanism is perceived workplace harassment (eg, bullying) by senior physicians and/or patients that is linked to the development of PTSD and increased globally to health care professionals during the pandemic.^87^ The risk of PTSD may increase throughout training as more cross-sectional studies showing higher PTSD rates among more senior residents and early-career attendings.^88^ These results suggest that there may be a range of time during residency and transition to a practicing attending that physicians were more prone to PTSD. The longer work hours by resident physicians compared with attendings may have lead to increased exposure to traumatic events and manifested into greater instances of PTSD development. Considering the paucity of literature analyzing PTSD in trainees and/or attendings, further research should be conducted to better elucidate such trends in both a general and COVID-19 context. Moreover, 8 of the 10 studies that stratified by age showed that older physicians had a higher prevalence of PTSD. This appears to conflict with the results of trainees relative to attendings; however, studies reporting age were highly variable in PTSD prevalence, precluding meta-analysis, so the impact of age remains less clear.

The prevalence of PTSD varied greatly between specialties, with the specialties of highest prevalence reported in emergency medicine and family medicine. Similarly, the COVID-19 pandemic produced many severe respiratory issues and related complications with primary responders often being emergency physicians, who subsequently had greater opportunities to witness patient deaths. Family physicians may represent another first-line health care professional for those with COVID-19 and long-term pandemic-induced trauma and health concerns.^89^ The majority of studies focused on individual specialties with few comparing across specialties ranged.

### Limitations

This study has limitations. First, almost half of the studies did not report response rates, and of those that did, some did not explicitly stratify the response rate for physicians as they had a broad health care population. Low or unreported response rates limit generalizability. Second, few studies examined changes before and during the pandemic in the physician population relative to the general population. This complicates the ability to disentangle a physician-specific change in PTSD from changes COVID-19–related impacts in the general population. Third, the lack of consistent scales and cutoffs introduced heterogeneity between studies, limited the ability to compare studies and determine a true prevalence estimate. Fourth, there were only 2 longitudinal studies retrieved that made it hard to analyze how PTSD prevalence changed throughout the course of the COVID-19 pandemic or from before the pandemic, which would have more effectively highlighted the role a pandemic could have on developing PTSD. Fifth, self-report scales were used in the included observational studies as opposed to clinician-administered diagnostic questionnaires due to greater feasibility; however, this compromises the accuracy of the data. Sixth, our definition of PTSD overlapped with another clinically similar phenomenon known as adjustment disorder. The difference between PTSD and adjustment disorder is that the former requires at least 6 months of persistent PTSD symptoms while adjustment disorder is more temporary. Because we could not discern the timeline of physician PTSD symptoms, the reported prevalence of PTSD may be tainted by instances of adjustment disorder. Seventh, our review was limited to articles in English, which limits the representation of studies from other countries.

## Conclusions

The findings of this meta-analysis and systematic review suggest a pooled estimate of physician PTSD of 18% during the COVID-19 pandemic, a figure that is higher than PTSD in the general population or previously reported PTSD in physicians before the pandemic. Most studies were cross-sectional and survey-based, with a large degree of heterogeneity in the assessment tools used and defined cutoff scores. Females and trainees appear more susceptible to developing PTSD. The high prevalence of PTSD suggests that system level changes may be indicated to support physician health, which can include wellness supports and specific interventions to target and alleviate root causes. Furthermore, additional research regarding physician PTSD in general, such as studies temporally unrelated to the COVID-19 pandemic, would better help characterize physician PTSD in typical health care climates. Such research can highlight any variances in risk factors among physicians who were and were not impacted by the COVID-19 pandemic, which can better shed light on how the pandemic influenced physician wellness.

## Notes

Supplement 1. **eAppendix 1.** Detailed Search Strategy **eAppendix 2.** Additional Data Methodology: Protocol, Data Selection and Extraction, Data Synthesis **eTable 1.** Studies Assessing PTSD in Physicians by Specialty Type and/or Career Stage **eTable 2.** Newcastle Ottawa Scale (NOS) Rating of Included Studies **eFigure 1.** Prevalence of PTSD Among Physicians During COVID-19: Study of Sample Size Over 200 **eFigure 2.** Prevalence of PTSD Among Physicians During COVID-19: Response Rate of at Least 50% **eFigure 3.** Prevalence of PTSD Among Physicians During COVID-19 by Continent **eFigure 4.** Prevalence of PTSD Among Physicians During COVID-19 by PTSD Scale **eFigure 5.** Prevalence of PTSD Among Physicians During COVID-19: Women vs. Men **eFigure 6.** Prevalence of PTSD Among Physicians During COVID-19: Low Risk of Bias **eFigure 7.** Prevalence of PTSD Among Physicians During COVID-19 by Among Physicians During COVID-19: Trainee vs Attendings **eFigure 8.** Prevalence of PTSD Among Physicians During COVID-19 by Medical Specialties

Supplement 2.
Data Sharing Statement
