# Supplementary material for: Physician Posttraumatic Stress Disorder During COVID-19: A Systematic Review and Meta-Analysis
Source: JAMA Netw Open. 2024 Jul 24;7(7):e2423316. doi: 10.1001/jamanetworkopen.2024.23316 (PMC11270139; doi:10.1001/jamanetworkopen.2024.23316)
Supplement: Supplement 1. — eAppendix 1. Detailed Search Strategy eAppendix 2. Additional Data Methodology: Protocol, Data Selection and Extraction, Data Synthesis eTable 1. Studies Assessing PTSD in Physicians by Specialty Type and/or Career Stage eTable 2. Newcastle Ottawa Scale (NOS) Rating of Included Studies eFigure 1. Prevalence of PTSD Among Physicians During COVID-19: Study of Sample Size Over 200 eFigure 2. Prevalence of PTSD Among Physicians During COVID-19: Response Rate of at Least 50% eFigure 3. Prevalence of PTSD Among Physicians During COVID-19 by Continent eFigure 4. Prevalence of PTSD Among Physicians During COVID-19 by PTSD Scale eFigure 5. Prevalence of PTSD Among Physicians During COVID-19: Women vs. Men eFigure 6. Prevalence of PTSD Among Physicians During COVID-19: Low Risk of Bias eFigure 7. Prevalence of PTSD Among Physicians During COVID-19 by Among Physicians During COVID-19: Trainee vs Attendings eFigure 8. Prevalence of PTSD Among Physicians During COVID-19 by Medical Specialties [file jamanetwopen-e2423316-s001.pdf]

Supplemental Online Content

Kamra M, Dhaliwal S, Li W, et al. Physician posttraumatic stress disorder during the COVID-19 pandemic: a systematic review and meta-analysis. *JAMA Netw Open*. 2024. 7(7):e2423316. doi:10.1001/jamanetworkopen.2024.23316

eAppendix 1. Detailed Search Strategy

eAppendix 2. Additional Data Methodology: Protocol, Data Selection and Extraction, Data Synthesis

eTable 1. Studies Assessing PTSD in Physicians by Specialty Type and/or Career Stage

eTable 2. Newcastle Ottawa Scale (NOS) Rating of Included Studies

eFigure 1. Prevalence of PTSD Among Physicians During COVID-19: Study of Sample Size Over 200

eFigure 2. Prevalence of PTSD Among Physicians During COVID-19: Response Rate of at Least 50%

eFigure 3. Prevalence of PTSD Among Physicians During COVID-19 by Continent

eFigure 4. Prevalence of PTSD Among Physicians During COVID-19 by PTSD Scale

eFigure 5. Prevalence of PTSD Among Physicians During COVID-19: Low Risk of Bias

eFigure 6. Prevalence of PTSD Among Physicians During COVID-19: Women vs. Men

eFigure 7. Prevalence of PTSD Among Physicians During COVID-19 by Among Physicians During COVID-19: Trainee vs Attendings

eFigure 8. Prevalence of PTSD Among Physicians During COVID-19 by Medical Specialties

This supplemental material has been provided by the authors to give readers additional information about their work.

© 2024 Kamra M et al. *JAMA Network Open*.

eAppendix 1: Detailed Search Strategy

Completed November 12, 2022
Medline (OVID interface)
Date restriction: Limit ALL to dt=20100101-20200229

Physician Terms	PTSD Terms
MESH Terms:<br><br>Physicians/ or Cardiologists/ or Pulmonary Medicine/ or Internal Medicine/ or Pediatricians/ or Gynecology/ or Obstetrics/ or Orthopedic Surgeons/ or "Oral and Maxillofacial Surgeons"/ or Surgeons/ or Psychiatry/ or Dermatologists/ or Endocrinologists/ or Gastroenterologists/ or Nephrologists/ or Ophthalmology/ or Pulmonologists/ or Neurologists/ or Radiologists/ or Anesthesiologists/ or Oncologists/ or Neurosurgeons/ or Allergists/ or Physicians, Emergency/ or Pathologists/ or Physiatrists/ or Rheumatologists/ or Urologists/<br><br>Keywords:<br><br>(doctor* or physician* or cardiologist* or (Pulmonary adj Medicine) or (Internal adj Medicine) or P?ediatrician* or Gynecolog* or Obstetrics* or Surgeon* or Psychiatr* or Dermatologist* or Endocrinologist* or Gastroenterologist* or Nephrologist* or Ophthalmolog* or Neurologist* or Radiologist* or Anesthesiologist* or Oncologist* or Neurosurgeon* or Allergist* or (Family adj Medicine) or Pathologist* or Physiatrist* or Rheumatologist* or Urologist*).ti,ab,kf.	MESH Terms:<br><br>Stress Disorders, Traumatic, Acute/ or Stress Disorders, Post-Traumatic/<br><br>Keywords:<br><br>(PTSD or post?trauma* stress disorder* or post?trauma* stress or ((post?trauma* or PTSD) adj2 symptom*)).ti,ab,kf.

APA PsychInfo (OVID interface)
Date restriction: Limit ALL by YEAR – *end in 2020 and manually weed out Mar-Dec 2020*

Physician Terms	PTSD Terms
MESH Terms:<br><br>Physicians/ or Cardiologists/ or Pulmonary Medicine/ or Internal Medicine/ or Pediatricians/ or Gynecology/ or Obstetrics/ or Orthopedic Surgeons/ or "Oral and Maxillofacial Surgeons"/ or Surgeons/ or Psychiatry/ or Dermatologists/ or Endocrinologists/ or Gastroenterologists/ or Nephrologists/ or Ophthalmology/ or Pulmonologists/ or Neurologists/ or Radiologists/ or Anesthesiologists/ or Oncologists/ or Neurosurgeons/ or Allergists/ or Physicians, Emergency/ or Pathologists/ or Physiatrists/ or Rheumatologists/ or Urologists/<br><br>Keywords:	MESH Terms:<br><br>Anxiety/ or Anxiety Disorders/ or Stress Disorders, Post-Traumatic/<br><br>Keywords:<br><br>(PTSD or post?trauma* stress disorder* or post?trauma* stress or ((post?trauma* or PTSD) adj2 symptom*)).ti,ab,kf.

© 2024 Kamra M et al. *JAMA Network Open*.

(doctor* or physician* or cardiologist* or (Pulmonary adj Medicine) or (Internal adj Medicine) or P?ediatrician* or Gynecolog* or Obstetrics* or Surgeon* or Psychiatr* or Dermatologist* or Endocrinologist* or Gastroenterologist* or Nephrologist* or Ophthalmolog* or Neurologist* or Radiologist* or Anesthesiologist* or Oncologist* or Neurosurgeon* or Allergist* or (Family adj Medicine) or Pathologist* or Physiatrist* or Rheumatologist* or Urologist*).ti,ab,kf.	

EMBASE (OVID interface)
Date restriction: Limit ALL to dd=19900101-20200229

**Physician Terms**	**PTSD Terms**
MESH Terms:<br><br>Physicians/ or Cardiologists/ or Pulmonary Medicine/ or Internal Medicine/ or Pediatricians/ or Gynecology/ or Obstetrics/ or Orthopedic Surgeons/ or "Oral and Maxillofacial Surgeons"/ or Surgeons/ or Psychiatry/ or Dermatologists/ or Endocrinologists/ or Gastroenterologists/ or Nephrologists/ or Ophthalmology/ or Pulmonologists/ or Neurologists/ or Radiologists/ or Anesthesiologists/ or Oncologists/ or Neurosurgeons/ or Allergists/ or Physicians, Emergency/ or Pathologists/ or Physiatrists/ or Rheumatologists/ or Urologists/<br><br>Keywords:<br><br>(doctor* or physician* or cardiologist* or (Pulmonary adj Medicine) or (Internal adj Medicine) or P?ediatrician* or Gynecolog* or Obstetrics* or Surgeon* or Psychiatr* or Dermatologist* or Endocrinologist* or Gastroenterologist* or Nephrologist* or Ophthalmolog* or Neurologist* or Radiologist* or Anesthesiologist* or Oncologist* or Neurosurgeon* or Allergist* or (Family adj Medicine) or Pathologist* or Physiatrist* or Rheumatologist* or Urologist*).ti,ab,kf.	MESH Terms:<br><br>Anxiety/ or Anxiety Disorders/ or Stress Disorders, Post-Traumatic/<br><br>Keywords:<br><br>(PTSD or post?trauma* stress disorder* or post?trauma* stress or ((post?trauma* or PTSD) adj2 symptom*)).ti,ab,kf.

© 2024 Kamra M et al. *JAMA Network Open*.

eAppendix 2. Additional Data Methodology: Protocol, Data Selection and Extraction, Data Synthesis

Methods:

Protocol

We excluded studies that (1) examined only the prevalence of PTSD in medical students or non-physician health care professionals (e.g., nurses, technicians, midwives, etc.); (2) included both physicians and non-physicians without reporting on both groups separately; (3) were not original articles (e.g., were comments, letters, or reviews); or (4) did not include or indicate COVID-19 as the setting of concern, even if the data was based during the pandemic period.

A literature search was conducted by consulting 3 databases: Ovid Medline, Embase, and PsycINFO. Keywords include 'physician' and various specialties of physicians (i.e. 'neurologist,' 'gynecologist,' 'surgeon,' etc.) as well as PTSD and its variants (i.e. 'post-traumatic stress disorder,' 'post-traumatic stress,' etc.) A detailed outline of the search strategy used can be found in the supplementary materials. It is important to note that the formal definition of PTSD would require persistent symptoms of at least 6 months, however, the precise timeline of physician PTSD history is not ascertainable from the literature. Thus, the persistency of symptoms measured in time will not be used to characterize the development of PTSD for this study.

Data Selection and Extraction

All relevant articles were reviewed by two independent screeners for inclusion and categorized as eligible, ineligible, or possibly eligible. Conflicts were discussed and resolved involved screeners. Eligible articles had their full texts retrieved and reviewed for final inclusion based on consensus on the pair of reviewers. The Covidence Systematic Review Management Software (Covidence, Melbourne, VIC, Australia) was used for screening. Following full-text screening, two reviewers independently extracted data from included full-text articles. Data discrepancies were resolved between the reviewers. The following information was extracted: descriptive statistics related to study location, year and design, as well as participant age, sex,

© 2024 Kamra M et al. *JAMA Network Open.*

career stage (i.e. postgraduate, resident, and attending physician), specialty; the number of individuals who screened positive for PTSD or PTSD symptoms; any subgroup assessments where the results were stratified by sex, age, career stage, and/or specialty; the method of assessment for PTSD (i.e. IES-R scale, PC-PTSD scale, etc.); and cut-off scores for a positive assessment of PTSD.

Data Methodology: Data Synthesis

The prevalence of PTSD from 57 studies was pooled and analyzed for heterogeneity. For meta-analyses, the results of each study were treated as dichotomous variables with any individual with PTSD or PTSD symptoms above a clinical threshold considered as a positive screen. Based on the large degree of heterogeneity, a Baujat plot was conducted to see if the omission of any studies would mitigate any heterogeneity.[1] A random effects model was subsequently employed to account for the heterogeneity when constructing forest plots and odds ratios (OR). Pooled results were calculated for all included studies and subgroups of sex, specialty, and career stage via a random intercept logistic regression model. [1,2] The model incorporated a maximum-likelihood estimator for τ^2, a Logit transformation, and a Clopper-Pearson confidence interval estimate for individual studies. To calculate pooled OR estimates, the Mantel-Haenszel method was used to determine the weight of each study and Knapp-Hartung adjustments were made for the random effects model.[3] For prospective cohort and longitudinal studies which reported proportional estimates throughout the course of the study, the average prevalence across the surveys was used. Further, prevalence of each study was stratified by year(s) of data collection to understand how the development of PTSD symptoms changed throughout the pandemic. Heterogeneity was assessed by the I^2 statistic. Results were presented as forest plots. All analyses were conducted using R version 4.2.2 (R Foundation for Statistical Computing) with RStudio. All statistical tests were 2-sided at the alpha level of <0.05. Further, sensitivity analyses were done to account for sample size, response rate, PTSD scale, risk of bias, and geographical location (continent).

© 2024 Kamra M et al. *JAMA Network Open.*

eTable 1: Studies assessing PTSD in physicians by specialty type and/or career stage.

Author, Year	Outcome Assessment	Definition of Outcome	Specialty Distribution	Outcome n (%)	Stage Distribution	Outcome n (%)
Udgiri et al, 2021[4]	IES-R	Moderate (≥ 33) is considered the best cut-off for probable PTSD diagnosis	N/A	N/A	PG I: 37<br>PG II: 26<br>PG III: 17	Mild (PG I): 23 (62.2)<br>Mild (PG II): 10 (38.5)<br>Mild (PG III): 9 (52.9)<br><br>Moderate (PG I): 7 (18.9)<br>Moderate (PG II): 8 (30.8)<br>Moderate (PG III): 1 (5.9)<br><br>Severe (PG I): 3 (8.1)<br>Severe (PG II): 1 (3.8)<br>Severe (PG III): 5 (29.4)
Pascoe et al, 2022[5]	IES-R	Moderate to Severe: >9			Junior Doctor: 745<br>Senior Doctor: 1221	Junior Doctor: 328 (44.2)<br>Senior Doctor: 377 (31.0)
Pazmino Erazo et al, 2021[6]	IES-R	Mild: 9–25<br>Moderate: 26–43<br>Severe: >44<br><br>For this review, cut-off will be considered moderate score (≥ 26)			Postgraduate: 166<br>Resident: 212<br>Treating Doctor: 179	Postgraduate (Mild): 51 (30.7)<br>Postgraduate (Moderate): 47 (28.3)<br>Postgraduate (Severe): 35 (21.1)<br><br>Resident (Mild): 72 (33.9)<br>Resident (Moderate): 54 (25.6)<br>Resident (Severe): 38 (17.9)<br><br>Treating Doctor (Mild): 50 (27.9)<br>Treating Doctor (Moderate): 38 (21.2)

© 2024 Kamra M et al. *JAMA Network Open*.

						Treating Doctor (Severe): 38 (21.2)
Pasin et al, 2020[7]	IES-R	Probable Diagnosis for PTSD: ≥33	Anesthesia and Intensive Care: 485<br>Emergency Medicine: 75	Anesthesia and Intensive Care: 71 (14.6)<br>Emergency Medicine: 24 (32)	1st year residency: 113<br>2nd year residency: 117<br>3rd year residency: 79<br>4th year residency: 90<br>5th year residency: 101	1st year residency: 12 (10.6)<br>2nd year residency: 21 (17.9)<br>3rd year residency: 21 (26.6)<br>4th year residency: 24 (26.7)<br>5th year residency: 27 (26.7)
**Author, Year**	**Outcome Assessment**	**Definition of Outcome**	**Specialty Distribution**	**Outcome n (%)**	**Stage Distribution**	**Outcome n (%)**
Das et al, 2021[8]	IES-R	For this review, cutoff will be considered ≥33			Doctor consultant: 40<br>Senior resident: 20<br>Postgraduate: 126<br>Intern: 117	Clinical concern for PTSD (Doctor consultant): 16 (40.0)<br>Clinical concern for PTSD (Senior resident): 1 (5.0)<br>Clinical concern for PTSD (Postgraduate): 13 (10.3)<br>Clinical concern for PTSD (Intern): 20 (17.1)<br><br>Probable PTSD (Doctor consultant): 3 (7.5)<br>Probable PTSD (Senior resident): 2 (10.0)<br>Probable PTSD (Postgraduate): 9 (7.1)<br>Probable PTSD (Intern): 12 (10.3)<br><br>Definitive PTSD (Doctor consultant): 8 (20.0)

© 2024 Kamra M et al. *JAMA Network Open*.

						Definitive PTSD (Senior resident): 10 (50.0)<br>Definitive PTSD (Postgraduate): 41 (32.5)<br>Definitive PTSD (Intern): 48 (41.0)
Roberts et al, 2021[9]	IES-R	Probable PTSD: >33	Emergency Medicine: 2005<br>Anaesthetics: 2955<br>Intensive Care Medicine: 920	Emergency Medicine (>24): 176 (8.8)<br>Emergency Medicine (>33): 109 (5.4)<br>Anaesthetics (>24): 142 (4.8)<br>Anaesthetics (>33): 78 (2.6)<br>Intensive Care Medicine (>24): 44 (4.8)<br>Intensive Care Medicine (>33): 22 (2.4)		
**Author, Year**	**Outcome Assessment**	**Definition of Outcome**	**Specialty Distribution**	**Outcome n (%)**	**Stage Distribution**	**Outcome n (%)**
Tan et al, 2021[10]	IES-R	Cut-off implied: ≥44			Trainee: 1156<br>Consultant (Attending): 2040	Trainee: 290 (25.1)<br>Consultant (Attending): 463 (22.7)
Lasalvia et al, 2021[11]	IES-R	Clinical PTSD: ≥24			April – May 2020:<br>Physician: 169<br>Resident: 190	April – May 2020:<br>Physician: 72 (42.6)<br>Resident: 69 (36.3)

© 2024 Kamra M et al. *JAMA Network Open*.

					<u>April – May 2021:</u><br>Physician: 50<br>Resident: 52	<u>April – May 2021:</u><br>Physician: 32 (64.0)<br>Resident: 34 (65.4)
Azoulay et al, 2021[12]	IES-R	Symptoms of PTSD: ≥26			Residents and Interns: 97<br>Attending Physicians: 175	Residents and Interns: 26 (26.8)<br>Attending Physicians: 38 (21.7)
Civantos et al, 2020[13]	IES-R	For this review, PTSD cut-off assumed to be considered the moderate score (≥ 26)	Otolaryngologist: 349	<u>Point Prevalence:</u><br>Mild: 114 (32.7)<br>Moderate: 73 (20.9)<br>Severe: 23 (6.6)	Resident: 165 (47.3)<br>Attending: 184 (52.7)	Mild:<br>Resident: 47 (28.5)<br>Attending: 67 (36.4)<br><br>Moderate:<br>Resident: 39 (23.6)<br>Attending: 34 (18.5)<br><br>Severe:<br>Resident: 10 (6.1)<br>Attending: 10 (7.1)
Gainer et al, 2021[14]	APCL	Clinically Meaningful Threshold for PTSD: ≥14	Pediatrics: 270 (15.7)<br>Emergency Medicine: 192 (11.2)<br>Internal Medicine: 177 (10.3)<br>Family Medicine: 163 (9.5)<br>Psychiatry: 134 (7.8)<br>IM Specialties: 111 (6.5)	Pediatrics: 79 (29.5)<br>Emergency Medicine: 56 (29.1)<br>Internal Medicine: 48 (27.4)<br>Family Medicine: 51 (31.6)<br>Psychiatry: 22 (16.8)<br>IM Specialties: 34 (30.8)<br>ObGyn: 31 (38.5)<br>Neurology: 12 (14.7)	Trainees: 447 (26.2)<br>Non-trainees: 1261 (73.8)	Trainees: 137 (30.7)<br>Non-trainees: 333 (26.4)

© 2024 Kamra M et al. *JAMA Network Open*.

			ObGyn: 82 (4.8)<br>Neurology: 79 (4.6)<br>Pulmonary/Critical Care: 70 (4.1)<br>Anesthesiology: 66 (3.8)<br>Surgery: 60 (3.5)<br>Pathology: 54 (3.1)<br>Radiology/Nuclear Medicine: 53 (3.1)<br>Infectious Diseases: 39 (2.3)<br>Other*: 167 (9.7)<br><br>*Allergy & Immunology, Dermatology, Physical Medicine & Rehabilitation, Medical Genetics and Genomics, Preventive Medicine/Public Health, Ophthalmology, Otolaryngology, Urology	Pulmonary/Critical Care: 19 (27.3)<br>Anesthesiology: 14 (21.0)<br>Surgery: 21 (35.1)<br>Pathology: 14 (26.9)<br>Radiology/Nuclear Medicine: 13 (25.5)<br>Infectious Diseases: 9 (23.7)<br>Other*: 45 (26.8)<br><br>*Allergy & Immunology, Dermatology, Physical Medicine & Rehabilitation, Medical Genetics and Genomics, Preventive Medicine/Public Health, Ophthalmology, Otolaryngology, Urology		
**Author, Year**	**Outcome Assessment**	**Definition of Outcome**	**Specialty Distribution**	**Outcome n (%)**	**Stage Distribution**	**Outcome n (%)**
Marco et al, 2020[15]	PCL-5	Symptoms suggestive of PTSD: ≥33	Emergency Medicine: 1300	290 (23.3)	Years practicing:<br><10 years: 344<br>>20 years: 497	Years practicing:<br><10 years: 89 (25.9)<br>>20 years: 96 (19.3)

© 2024 Kamra M et al. *JAMA Network Open*.

**Author, Year**	**Outcome Assessment**	**Definition of Outcome**	**Specialty Distribution**	**Outcome n (%)**	**Stage Distribution**	**Outcome n (%)**
Lombard et al, 2022[16]	PCL-5	Provisional diagnosis of PTSD: ≥33	Medical officer: 6<br>Registrar (still in training): 69<br>Diplomate anaesthetist: 27<br>Consultant anaesthetist: 288	Medical officer: 2 (33.3)<br>Registrar (still in training): 18 (26.1)<br>Diplomate anaesthetist: 4 (14.8)<br>Consultant anaesthetist: 44 (15.5)	Medical officer: 6<br>Registrar (still in training): 69<br>Diplomate anaesthetist: 27<br>Consultant anaesthetist: 288<br><br><5 years of experience: 35<br>5-9 years of experience: 94<br>10-15 years of experience: 79<br>>15 years of experience: 177	Medical officer: 2 (33.3)<br>Registrar (still in training): 18 (26.1)<br>Diplomate anaesthetist: 4 (14.8)<br>Consultant anaesthetist: 44 (15.5)<br><br><5 years of experience: 7 (20.0)<br>5-9 years of experience: 23 (24.5)<br>10-15 years of experience: 17 (21.5)<br>>15 years of experience: 20 (11.3)
Kaplan et al, 2021[17]	PCL4-5	Positive PTSD Screen: ≥8	Other medical speciality: 95<br>Internal medicine: 186<br>Surgical: 143<br>Hospital-based: 125	Other medical speciality: 10 (10.5)<br>Internal medicine: 31 (16.7)<br>Surgical: 14 (9.8)<br>Hospital-based: 15 (12.0)	<3 years in practice: 207<br>≥3 years in practice: 293	<3 years in practice: 24 (11.6)<br>≥3 years in practice: 37 (12.6)
Ouazzani et al, 2021[18]	PCL-5	Probable PTSD: ≥33	Generalist doctors: 186 Specialist: 242	Generalist doctors: 58 (31.8)<br>Specialist: 56 (23.1)	Intern doctors: 232<br>Resident doctors: 607<br>Attending doctors: 428<br><br><u>Years of experience</u>	Intern doctors: 52 (22.4)<br>Resident doctors: 110 (18.1)<br>Attending doctors: 114 (26.6)<br><br><u>Years of experience</u>

© 2024 Kamra M et al. *JAMA Network Open*.

					<2: 425<br>2-5: 503<br>6-10: 194<br>>10: 145	<2: 94 (22.1)<br>2-5: 102 (20.3)<br>6-10: 48 (24.7)<br>>10: 32 (22.1)
Piacentini et al, 2022[19]	PCL-5	Cut-off implied to be ≥40	Physicians in contact with patients and/or biological material: 808<br><br>Anaesthesiologists: 87	Physicians in contact with patients and/or biological material: 81 (10.0)<br><br>Anaesthesiologists: 19 (21.8)		
**Author, Year**	**Outcome Assessment**	**Definition of Outcome**	**Specialty Distribution**	**Outcome n (%)**	**Stage Distribution**	**Outcome n (%)**
Chang et al, 2021[20]	PCL-5	At-risk PTSD symptomatology: ≥ 31	Emergency Medicine: 31	Emergency Medicine: 11 (35.5)	Resident: 31	Resident: 11 (35.5)
Dehon et al, 2021[21]	PCL-5	Probable PTSD Diagnosis: ≥31	Emergency Medicine: 255	19 (7.5)		
Schwartz et al 2022[22]	PC-PTSD-5	Probable PTSD: ≥3			Attending: 476<br>Fellow: 33<br>Resident: 93	Attending: 100 (21.0)<br>Fellow: 9 (27.3)<br>Resident: 28 (30.1)
Guo et al, 2022[23]	PC-PTSD	Probable PTSD: ≥2	Anaesthesiologists: 427	89 (20.8)		
Baumann et al, 2021[24]	PC-PTSD	'Increased Risk for PTSD:' ≥ 3	Emergency Medicine<br><br>First survey: 426<br><br>Follow-up survey: 262	Initial survey: 134 (31.5)<br><br>Follow-up: 85 (32.4)	Initial Survey (Resident): 113<br>Initial Survey (Faculty): 146<br><br>Follow-up Survey (Resident): 109	Initial Survey (Resident): 47 (41.6)<br>Initial Survey (Faculty): 38 (26.0)<br><br>Follow-up Survey (Resident): 34 (31.2)

© 2024 Kamra M et al. *JAMA Network Open*.

					Follow-up Survey (Faculty): 146	Follow-up Survey (Faculty): 32 (21.9)

© 2024 Kamra M et al. *JAMA Network Open*.

eTable 2. Newcastle Ottawa Scale (NOS) rating of included studies.

© 2024 Kamra M et al. *JAMA Network Open*.

Author, Year	Representativeness of the sample	Sample size	Non-respondents	Ascertainment of PTSD	Quality of descriptive statistics reporting	Overall
Wang et al, 2020[25]	1	1	0	1	1	Low
Moderato et al, 2021[26]	1	1	0	1	1	Low
Martinez-Caballero et al, 2021[27]	1	0	0	1	1	Low
Udgiri et al, 2021[4]	0	0	0	1	1	High
Dykes et al, 2022[28]	0	0	0	1	1	High
Li et al, 2022[29]	1	0	0	1	1	Low
Gainer et al, 2021[14]	1	1	0	1	1	Low
She et al, 2022[30]	1	1	0	1	1	Low
Chang et al, 2021[20]	0	0	0	1	1	High
Martsenkovskyi et al, 2022[31]	1	1	0	1	1	Low
Pascoe et al, 2022[5]	1	1	0	1	1	Low
Brady et al, 2022[32]	1	0	0	1	1	Low
Gonzalez-Mesa et al, 2021[33]	1	1	0	1	1	Low
Baumann et al, 2021[24]	1	1	0	1	1	Low
Pazmino Erazo et al, 2021[6]	1	1	0	1	1	Low
Oz Tunçer et al, 2022[34]	1	1	0	1	1	Low

© 2024 Kamra M et al. *JAMA Network Open*.

Holzer et al, 2017[35]	0	1	0	1	1	Low
Meena et al, 2022[36]	0	0	0	1	1	High
**Author, Year**	**Representativeness of the sample**	**Sample size**	**Non-respondents**	**Ascertainment of PTSD**	**Quality of descriptive statistics reporting**	**Overall**
Gileen et al, 2021[37]	1	1	0	1	1	Low
Chatzittofis et al, 2021[38]	1	0	0	1	1	Low
Hendrickson et al, 2021[39]	1	1	0	1	1	Low
Civantos et al, 2020[13]	1	1	0	1	1	Low
Ahmed et al, 2022[40]	0	0	0	1	1	High
Villalba-Arias et al, 2021[41]	1	1	0	1	1	Low
Greenberg et al, 2021[42]	1	1	0	1	1	Low
Leon Rojas et al, 2022[43]	1	1	0	1	1	Low
Pasin et al, 2020[7]	1	1	0	1	1	Low
Kader et al, 2021[44]	0	0	0	1	1	High
Das et al, 2021[8]	1	1	0	1	1	Low
Kalyanaraman et al, 2021[45]	1	1	0	1	1	Low
Marco et al, 2020[15]	1	1	0	1	1	Low

© 2024 Kamra M et al. *JAMA Network Open*.

Bahadirli et al, 2021[46]	1	1	0	1	1	Low
Costantini et al 2021[47]	0	0	0	1	1	High
Lombard et al, 2022[16]	1	1	0	1	1	Low
Guo et al, 2021[48]	1	1	1	1	1	Low
**Author, Year**	**Representativeness of the sample**	**Sample size**	**Non-respondents**	**Ascertainment of PTSD**	**Quality of descriptive statistics reporting**	**Overall**
Asnakew et al, 2021[49]	1	0	0	1	1	Low
Yang et al, 2022[50]	1	1	0	1	1	Low
Gorini et al, 2022[51]	1	1	0	1	1	Low
Kaplan et al, 2021[17]	0	1	0	1	1	Low
Roberts et al, 2021[9]	1	1	1	1	1	Low
Guo et al, 2022[23]	1	1	0	1	1	Low
Tan et al, 2021[10]	1	1	0	1	1	Low
Bates et al, 2021[52]	0	0	0	1	1	High
Lasalvia et al, 2021[11]	0	1	0	1	1	Low
Huang et al, 2021[53]	1	1	0	1	1	Low
Dehon et al, 2021[21]	1	1	1	1	1	Low

© 2024 Kamra M et al. *JAMA Network Open*.

Stafseth et al, 2022[54]	1	0	0	1	1	Low
Azoulay et al, 2021[12]	1	1	0	1	1	Low
Mosheva et al, 2021[55]	0	1	1	1	1	Low
Machado et al, 2022[56]	1	1	0	1	1	Low
Schwartz et al, 2022[22]	1	1	0	1	1	Low
Mehta et al, 2022[57]	1	0	0	1	1	Low
**Author, Year**	**Representativeness of the sample**	**Sample size**	**Non-respondents**	**Ascertainment of PTSD**	**Quality of descriptive statistics reporting**	**Overall**
Isik et al, 2021[58]	1	1	0	1	1	Low
Ouazzani et al, 2021[18]	1	1	0	1	1	Low
Piacentini et al, 2022[19]	1	1	0	1	0	Low
Seifeldin et al, 2022[59]	1	0	0	1	1	Low
Real-Ramirez et al, 2020[60]	1	0	0	1	1	Low

© 2024 Kamra M et al. *JAMA Network Open*.

eFigure 1. Prevalence of PTSD among physicians during COVID-19: Study of sample size over 200.

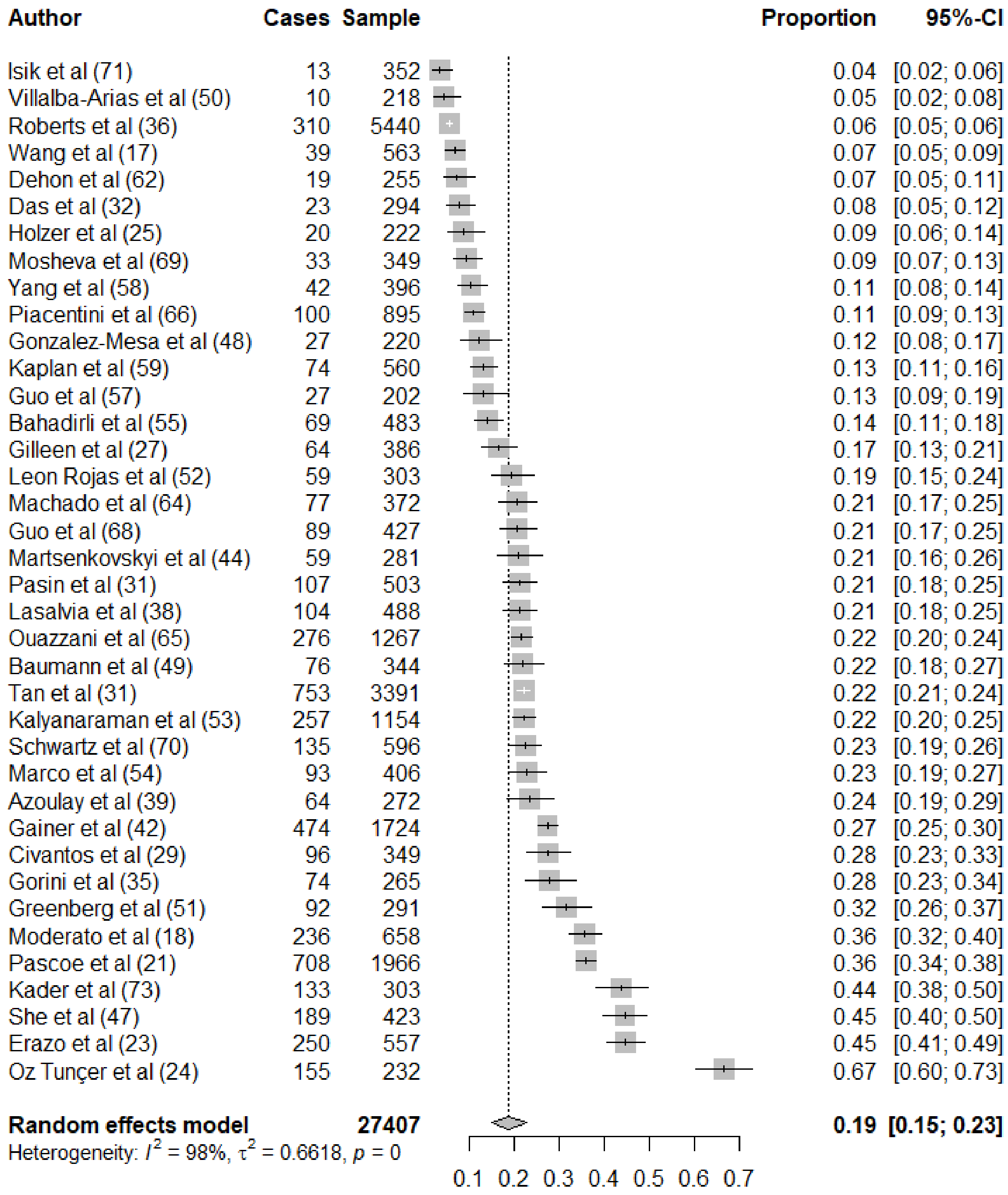

© 2024 Kamra M et al. *JAMA Network Open*.

eFigure 2. Prevalence of PTSD among physicians during COVID-19: Response rate of at least 50%.

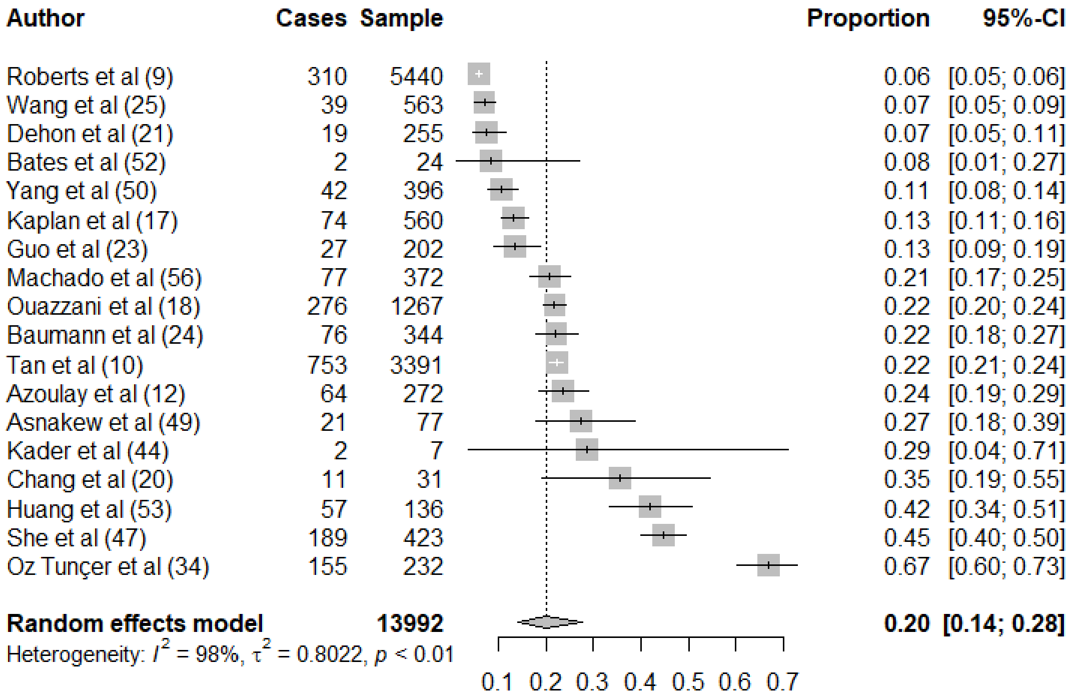

© 2024 Kamra M et al. *JAMA Network Open*.

eFigure 3. Prevalence of PTSD among physicians during COVID-19 by continent.

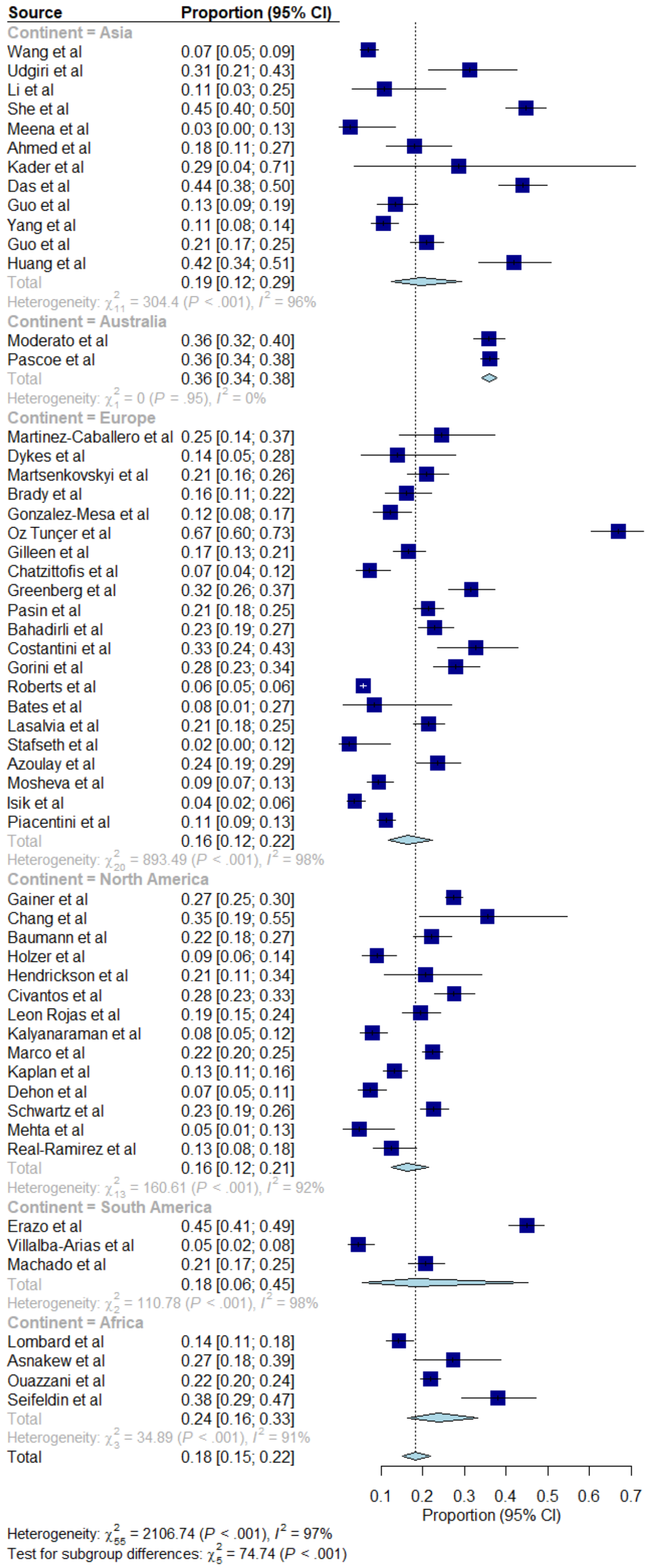

© 2024 Kamra M et al. *JAMA Network Open*.

eFigure 4. Prevalence of PTSD among physicians during COVID-19 by PTSD scale.

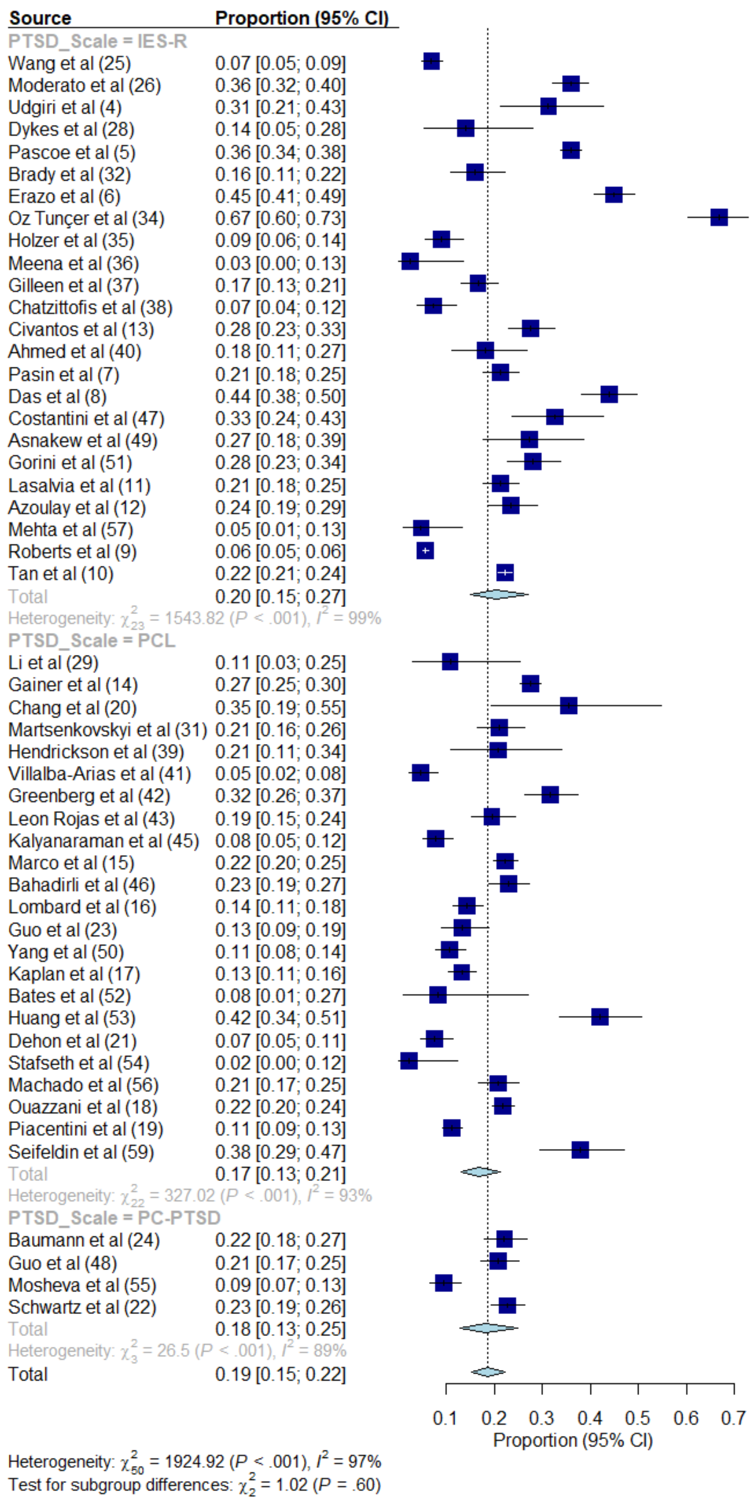

© 2024 Kamra M et al. *JAMA Network Open*.

eFigure 5. Prevalence of PTSD among physicians during COVID-19: Women vs. Men.

Source	OR (95% CI)
Moderato et al (26)	2.20 [1.35; 3.57]
Udgiri et al (4)	1.14 [0.44; 2.95]
Gainer et al (14)	2.28 [1.82; 2.86]
Gonzalez-Mesa et al (33)	3.01 [1.00; 9.07]
Baumann et al (24)	2.65 [1.51; 4.66]
Civantos et al (13)	2.20 [1.36; 3.55]
Pasin et al (7)	2.44 [1.53; 3.91]
Marco et al (15)	1.11 [0.84; 1.48]
Lombard et al (16)	2.53 [1.46; 4.41]
Kaplan et al (17)	0.99 [0.60; 1.63]
Schwartz et al (22)	2.24 [1.51; 3.34]
Ouazzani et al (18)	1.80 [1.35; 2.40]
Seifeldin et al (59)	2.93 [1.27; 6.71]
Total	1.93 [1.56; 2.39]

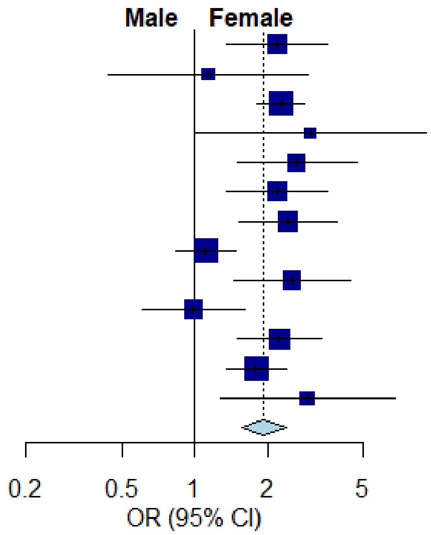

Heterogeneity: χ^2_{12} = 30.61 (P = .002), I^2 = 61%

© 2024 Kamra M et al. *JAMA Network Open*.

eFigure 6. Prevalence of PTSD among physicians during COVID-19: Low risk of bias.

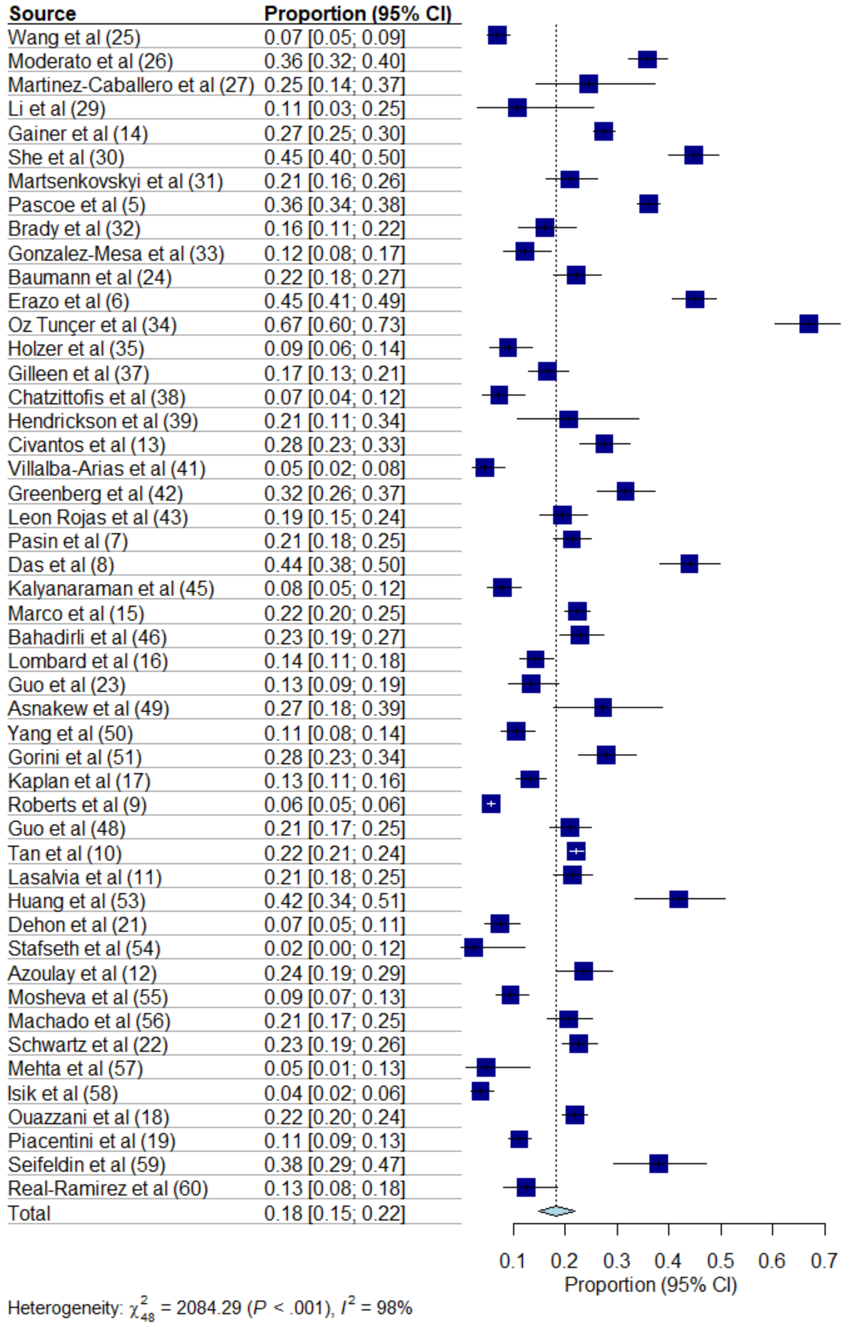

© 2024 Kamra M et al. *JAMA Network Open*.

eFigure 7. Prevalence of PTSD among physicians during COVID-19: Trainee vs. attendings

Source	OR (95% CI)
Gainer et al (14)	1.23 [0.97; 1.56]
Baumann et al (24)	1.80 [1.23; 2.63]
Pascoe et al (5)	1.76 [1.46; 2.13]
Erazo et al (6)	1.16 [0.81; 1.65]
Tan et al (10)	1.14 [0.96; 1.35]
Das et al (8)	1.33 [0.75; 2.37]
Lasalvia et al (11)	0.69 [0.45; 1.05]
Azoulay et al (39)	1.32 [0.74; 2.35]
Schwartz et al (22)	1.56 [1.00; 2.43]
Ouazzani et al (18)	1.31 [0.90; 1.89]
Civantos et al (13)	1.34 [0.84; 2.16]
Lombard et al (16)	1.91 [1.03; 3.54]
Total	1.33 [1.12; 1.57]

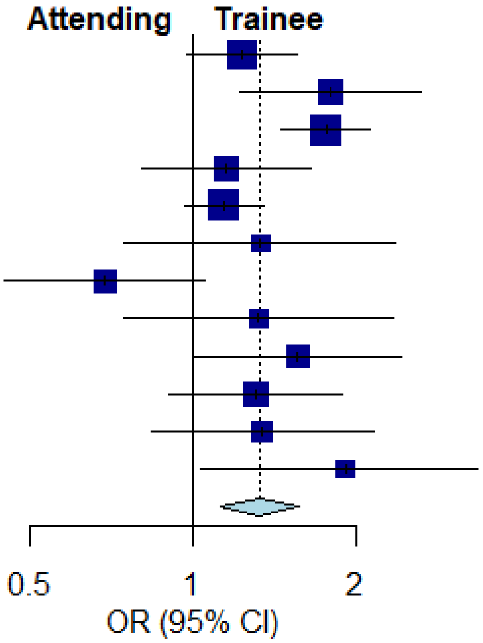

Heterogeneity: $\chi^2_{11} = 26.03$ (P = .006), I^2 = 58%

© 2024 Kamra M et al. *JAMA Network Open*.

eFigure 8. Prevalence of PTSD among physicians during COVID-19 by medical specialities

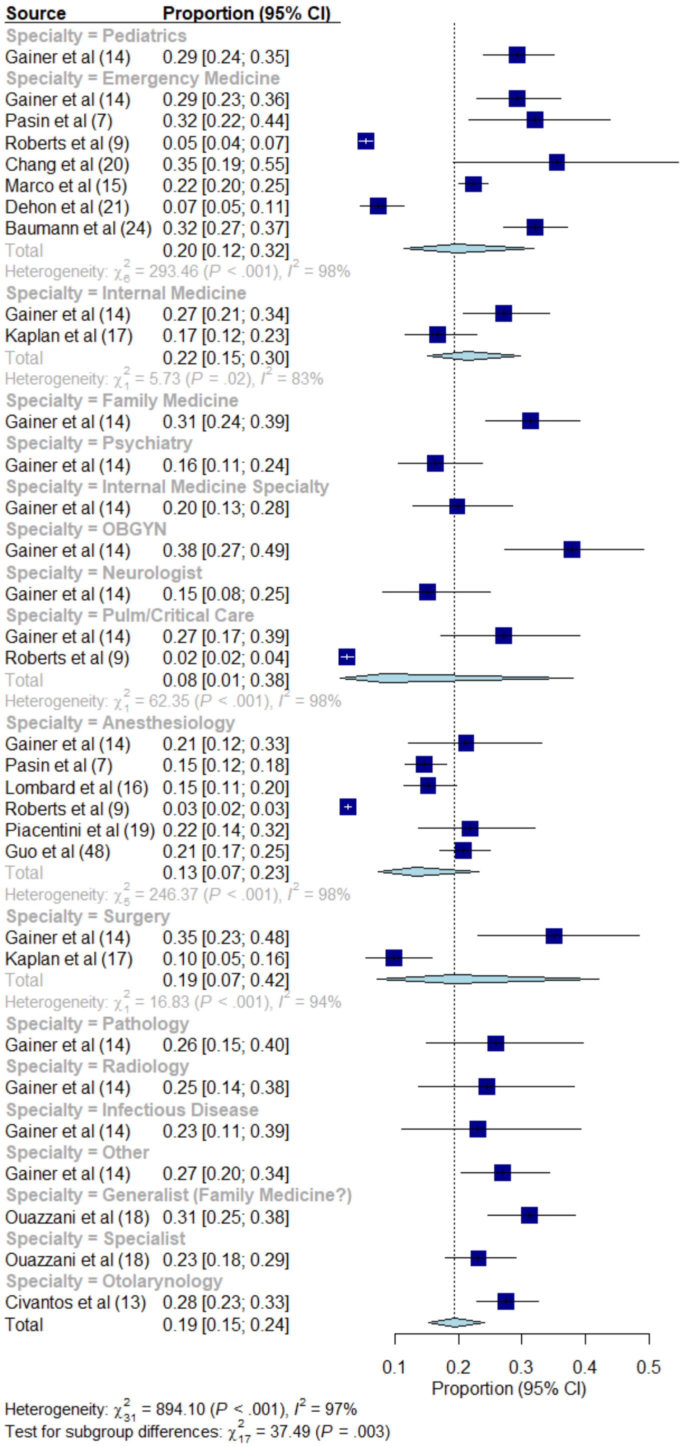

© 2024 Kamra M et al. *JAMA Network Open*.

References

1. Baujat B, Mahé C, Pignon J-P, et al. A graphical method for exploring heterogeneity in meta-analyses: application to a meta-analysis of 65 trials. Statistics in Medicine 2002;21(18):2641-52. doi: https://doi.org/10.1002/sim.1221

2. Stijnen T, Hamza TH, Özdemir P. Random effects meta-analysis of event outcome in the framework of the generalized linear mixed model with applications in sparse data. Stat Med. 2010;29 (29):3046-3067. doi:10.1002/sim.4040

3. Suurmond R, van Rhee H, Hak T. Introduction, comparison, and validation of Meta-Essentials: A free and simple tool for meta-analysis. Research Synthesis Methods 2017;8(4):537-53. doi: https://doi.org/10.1002/jrsm.1260

4. Udgiri RS, Biradar SG, Shannawaz M. Udgiri, R. S.; Biradar, S. G.; Shannawaz, M.. Journal of the Indian Medical Association 2021;4(119):19-23.

5. Pascoe A, Johnson D, Putland M, et al. Differential Impacts of the COVID-19 Pandemic on Mental Health Symptoms and Working Conditions for Senior and Junior Doctors in Australian Hospitals. J Occup Environ Med 2022;64(5):e291-e99. doi: 10.1097/jom.0000000000002505 [published Online First: 20220201]

6. Erazo EE, Alvear Velásquez MJ, Saltos Chávez IG, et al. Factors associated with psychiatric adverse effects in healthcare personnel during the COVID-19 pandemic in Ecuador. Rev Colomb Psiquiatr (Engl Ed) 2021;50(3):166-75. doi: 10.1016/j.rcpeng.2020.12.001 [published Online First: 20210810]

7. Pasin L, Sella N, Correale C, et al. Pandemic COVID-19: the residents' resilience. Acta Biomed 2020;91(4):e2020120. doi: 10.23750/abm.v91i4.10061 [published Online First: 20201001]

8. Das K, Ryali V, Bhavyasree R, et al. Postexposure psychological sequelae in frontline health workers to COVID-19 in Andhra Pradesh, India. Ind Psychiatry J 2021;30(1):123-30. doi: 10.4103/ipj.ipj_15_21 [published Online First: 20210630]

9. Roberts T, Daniels J, Hulme W, et al. Psychological distress and trauma in doctors providing frontline care during the COVID-19 pandemic in the United Kingdom and Ireland: a prospective longitudinal survey cohort study. BMJ Open 2021;11(7):e049680. doi: 10.1136/bmjopen-2021-049680 [published Online First: 20210709]

10. Tan YQ, Wang Z, Yap QV, et al. Psychological Health of Surgeons in a Time of COVID-19: A Global Survey. Ann Surg 2023;277(1):50-56. doi: 10.1097/sla.0000000000004775 [published Online First: 20210122]

11. Lasalvia A, Bodini L, Amaddeo F, et al. The Sustained Psychological Impact of the COVID-19 Pandemic on Health Care Workers One Year after the Outbreak-A Repeated Cross-Sectional Survey in a Tertiary Hospital of North-East Italy. Int J Environ Res Public Health 2021;18(24) doi: 10.3390/ijerph182413374 [published Online First: 20211219]12. Azoulay

© 2024 Kamra M et al. *JAMA Network Open*.

13. Civantos AM, Byrnes Y, Chang C, et al. Mental health among otolaryngology resident and attending physicians during the COVID-19 pandemic: National study. Head Neck 2020;42(7):1597-609. doi: 10.1002/hed.26292 [published Online First: 20200604]

14. Gainer DM, Nahhas RW, Bhatt NV, et al. Association Between Proportion of Workday Treating COVID-19 and Depression, Anxiety, and PTSD Outcomes in US Physicians. J Occup Environ Med 2021;63(2):89-97. doi: 10.1097/jom.0000000000002086

15. Marco CA, Larkin GL, Feeser VR, et al. Post-traumatic stress and stress disorders during the COVID-19 pandemic: Survey of emergency physicians. J Am Coll Emerg Physicians Open 2020;1(6):1594-601. doi: 10.1002/emp2.12305 [published Online First: 20201102]

16. Lombard T, Spijkerman S, Rooyen Cv. Prevalence and predisposing factors of post-traumatic stress symptoms in anaesthetists during the second wave of COVID-19 in South Africa. Southern African Journal of Anaesthesia and Analgesia 2022;28(2):62-68. doi: doi:10.36303/SAJAA.2022.28.2.2764

17. Kaplan CA, Chan CC, Feingold JH, et al. Psychological Consequences Among Residents and Fellows During the COVID-19 Pandemic in New York City: Implications for Targeted Interventions. Acad Med 2021;96(12):1722-31. doi: 10.1097/acm.0000000000004362

18. Ouazzani Housni Touhami Y, Maiouak M, Ouraghene A, et al. The prevalence and associated factors of depression, anxiety, and PTSD among Moroccan medical doctors in the COVID-19 pandemic: a national study. Psychol Health Med 2023;28(1):211-24. doi: 10.1080/13548506.2022.2067574 [published Online First: 20220427]

19. Piacentini SH, Tramacere I, Prioni S. The risk of post-traumatic stress disorder (PTSD) in Italian Highly Specialized Research Hospitals staff within two months of the pandemic declaration: an on-line survey. Ann Ist Super Sanita 2022;58(3):154-61. doi: 10.4415/ann_22_03_02

20. Chang J, Ray JM, Joseph D, et al. Burnout and Post-traumatic Stress Disorder Symptoms Among Emergency Medicine Resident Physicians During the COVID-19 Pandemic. West J Emerg Med 2022;23(2):251-57. doi: 10.5811/westjem.2021.11.53186 [published Online First: 20220228]

21. Dehon E, Zachrison KS, Peltzer-Jones J, et al. Sources of Distress and Coping Strategies Among Emergency Physicians During COVID-19. West J Emerg Med 2021;22(6):1240-52. doi: 10.5811/westjem.2021.9.53406 [published Online First: 20211027]

22. Schwartz RM, McCann-Pineo M, Bellehsen M, et al. The Impact of Physicians' COVID-19 Pandemic Occupational Experiences on Mental Health. J Occup Environ Med 2022;64(2):151-57. doi: 10.1097/jom.0000000000002380

23. Guo W-P, Min Q, Gu W-W, et al. Prevalence of mental health problems in frontline healthcare workers after the first outbreak of COVID-19 in China: a cross-sectional study. Health and Quality of Life Outcomes 2021;19(1):103. doi: 10.1186/s12955-021-01743-7

24. Baumann BM, Cooper RJ, Medak AJ, et al. Emergency physician stressors, concerns, and behavioral changes during COVID-19: A longitudinal study. Acad Emerg Med 2021;28(3):314-24. doi: 10.1111/acem.14219 [published Online First: 20210216]

© 2024 Kamra M et al. *JAMA Network Open*.

25. Wang Y, Ma S, Yang C, et al. Acute psychological effects of Coronavirus Disease 2019 outbreak among healthcare workers in China: a cross-sectional study. Translational Psychiatry 2020;10(1):348. doi: 10.1038/s41398-020-01031-w

26. Moderato L, Lazzeroni D, Oppo A, et al. Acute Stress Response Profiles in Health Workers Facing SARS-CoV-2. Frontiers in Psychology 2021;12 doi: 10.3389/fpsyg.2021.660156

27. Martínez-Caballero CM, Cárdaba-García RM, Varas-Manovel R, et al. Analyzing the Impact of COVID-19 Trauma on Developing Post-Traumatic Stress Disorder among Emergency Medical Workers in Spain. Int J Environ Res Public Health 2021;18(17) doi: 10.3390/ijerph18179132 [published Online First: 20210830]

28. Dykes N, Johnson O, Bamford P. Assessing the psychological impact of COVID-19 on intensive care workers: A single-centre cross-sectional UK-based study. J Intensive Care Soc 2022;23(2):132-38. doi: 10.1177/1751143720983182 [published Online First: 20210105]

29. Li M, Yu X, Wang D, et al. Association among resilience, post-traumatic stress disorder, and somatization in frontline healthcare workers in COVID-19: The mediating role of perceived stress. Front Psychiatry 2022;13:909071. doi: 10.3389/fpsyt.2022.909071 [published Online First: 20220916]

30. She R, Li L, Yang Q, et al. Associations between COVID-19 Work-Related Stressors and Posttraumatic Stress Symptoms among Chinese Doctors and Nurses: Application of Stress-Coping Theory. Int J Environ Res Public Health 2022;19(10) doi: 10.3390/ijerph19106201 [published Online First: 20220519]

31. Martsenkovskyi D, Babych V, Martsenkovska I, et al. Depression, anxiety, stress and trauma-related symptoms and their association with perceived social support in medical professionals during the COVID-19 pandemic in Ukraine. Advances in Psychiatry and Neurology/Postępy Psychiatrii i Neurologii 2022;31(1):6-14. doi: 10.5114/ppn.2022.11465732. Brady et al, 202222

33. González-Mesa E, Jiménez-López JS, Blasco-Alonso M, et al. Effects of SARS-CoV-2 Pandemic on the Mental Health of Spanish Ob-Gyn Specialists-A Nationwide Study. J Clin Med 2021;10(24) doi: 10.3390/jcm10245899 [published Online First: 20211215]

34. Öz Tunçer G, Dolu MH, Aydin S, et al. How did the COVID-19 Pandemic Affect Pediatric Neurologists? Pediatr Neurol 2022;132:41-44. doi: 10.1016/j.pediatrneurol.2022.05.003 [published Online First: 20220516]

35. Holzer KJ, Lou SS, Goss CW, et al. Impact of Changes in EHR Use during COVID-19 on Physician Trainee Mental Health. Appl Clin Inform 2021;12(3):507-17. doi: 10.1055/s-0041-1731000 [published Online First: 20210602]

36. Meena J, Dhiman S, Sharma R, et al. The Impact of the COVID-19 Pandemic on the Psychological Well-being of Healthcare Workers in Obstetrics and Gynaecology: An Observational Study at an Apex Institute. Cureus 2022;14(4):e24040. doi: 10.7759/cureus.24040 [published Online First: 20220411]

© 2024 Kamra M et al. *JAMA Network Open*.

37. Gilleen J, Santaolalla A, Valdearenas L, et al. Impact of the COVID-19 pandemic on the mental health and well-being of UK healthcare workers. BJPsych Open 2021;7(3):e88. doi: 10.1192/bjo.2021.42 [published Online First: 20210429]

38. Chatzittofis A, Karanikola M, Michailidou K, et al. Impact of the COVID-19 Pandemic on the Mental Health of Healthcare Workers. Int J Environ Res Public Health 2021;18(4) doi: 10.3390/ijerph18041435 [published Online First: 20210203]

39. Hendrickson RC, Slevin RA, Hoerster KD, et al. The Impact of the COVID-19 Pandemic on Mental Health, Occupational Functioning, and Professional Retention Among Health Care Workers and First Responders. J Gen Intern Med 2022;37(2):397-408. doi: 10.1007/s11606-021-07252-z [published Online First: 20211216]

40. Ahmed SIS, S. Y. M.; Nazneen, Z.; Bahadar, R.; Batool, A.; Ali, S.. Mental Health Consequences of Covid-19 among Health Care Workers in Abbottabad. Medical Forum Monthly 2022;33(2):48-52.

41. Villalba-Arias J, Estigarribia G, Bogado JA, et al. Mental health issues and psychological risk factors among Paraguayan healthcare workers during the COVID-19 pandemic. J Ment Health 2021:1-8. doi: 10.1080/09638237.2021.1979494 [published Online First: 20210929]

42. Greenberg N, Weston D, Hall C, et al. Mental health of staff working in intensive care during Covid-19. Occup Med (Lond) 2021;71(2):62-67. doi: 10.1093/occmed/kqaa220

43. León Rojas D, Castorena Torres F, Garza Ornelas B, et al. Mental health outcomes and risk factors among female physicians during the COVID-19 pandemic. Heliyon 2022;8(5):e09325. doi: 10.1016/j.heliyon.2022.e09325 [published Online First: 20220428]

44. Kader N, Elhusein B, Chandrappa NSK, et al. Perceived stress and post-traumatic stress disorder symptoms among intensive care unit staff caring for severely ill coronavirus disease 2019 patients during the pandemic: a national study. Ann Gen Psychiatry 2021;20(1):38. doi: 10.1186/s12991-021-00363-1 [published Online First: 20210821]

45. Kalyanaraman M, Sankar A, Timpo E, et al. Posttraumatic Stress among Pediatric Critical Care Physicians in the United States in Association with Coronavirus Disease 2019 Patient Care Experiences. J Intensive Care Med 2022;37(4):510-17. doi: 10.1177/08850666211059385 [published Online First: 20211123]

46. Bahadirli S, Sagaltici E. Post-traumatic stress disorder in healthcare workers of emergency departments during the pandemic: A cross-sectional study. Am J Emerg Med 2021;50:251-55. doi: 10.1016/j.ajem.2021.08.027 [published Online First: 20210814]

47. Costantini A, Mazzotti E, Cappitella C, et al. Prevalence and characteristics of distress in a sample of large hospital's workers in Rome in a period between two peaks of the covid-19 pandemic. Riv Psichiatr 2022;57(5):212-23. doi: 10.1708/3893.38744

48. Guo F, Han R, Luo T, et al. Psychological Distress Was Still Serious Among Anesthesiologists Under the Post COVID-19 Era. Psychol Res Behav Manag 2022;15:777-84. doi: 10.2147/prbm.S357566 [published Online First: 20220328]

© 2024 Kamra M et al. *JAMA Network Open*.

49. Asnakew S, Legas G, Muche Liyeh T, et al. Prevalence of post-traumatic stress disorder on health professionals in the era of COVID-19 pandemic, Northwest Ethiopia, 2020: A multi-centered cross-sectional study. PLoS One 2021;16(9):e0255340. doi: 10.1371/journal.pone.0255340 [published Online First: 20210914]

50. Yang Y, Liu D, Liu B, et al. Prevalence of Post-traumatic Stress Disorder Status Among Healthcare Workers and Its Impact on Their Mental Health During the Crisis of COVID-19: A Cross-Sectional Study. Front Public Health 2022;10:904550. doi: 10.3389/fpubh.2022.904550 [published Online First: 20220719]

51. Gorini A, Giuliani M, Fiabane E, et al. Prevalence of Psychopathological Symptoms and Their Determinants in Four Healthcare Workers' Categories during the Second Year of COVID-19 Pandemic. Int J Environ Res Public Health 2022;19(20) doi: 10.3390/ijerph192013712 [published Online First: 20221021]

52. Bates SM, Greer IA, Middeldorp S, et al. VTE, Thrombophilia, Antithrombotic Therapy, and Pregnancy: Antithrombotic Therapy and Prevention of Thrombosis, 9th ed: American College of Chest Physicians Evidence-Based Clinical Practice Guidelines. Chest 2012;141(2, Supplement):e691S-e736S. doi: https://doi.org/10.1378/chest.11-2300

53. Huang RW, Shen T, Ge LM, et al. Psychometric Properties of the Chinese Version of the Primary Care Post-Traumatic Stress Disorder Screen-5 for Medical Staff Exposed to the COVID-19 Pandemic. Psychol Res Behav Manag 2021;14:1371-78. doi: 10.2147/prbm.S329380 [published Online First: 20210902]

54. Stafseth SK, Skogstad L, Ræder J, et al. Symptoms of Anxiety, Depression, and Post-Traumatic Stress Disorder in Health Care Personnel in Norwegian ICUs during the First Wave of the COVID-19 Pandemic, a Prospective, Observational Cross-Sectional Study. Int J Environ Res Public Health 2022;19(12) doi: 10.3390/ijerph19127010 [published Online First: 20220608]

55. Mosheva M, Gross R, Hertz-Palmor N, et al. The association between witnessing patient death and mental health outcomes in frontline COVID-19 healthcare workers. Depress Anxiety 2021;38(4):468-79. doi: 10.1002/da.23140 [published Online First: 20210205]

56. Machado AV, Gonçalves RM, Gama CMF, et al. The different impacts of COVID-19 on the mental health of distinct health care worker categories. J Health Psychol 2023;28(5):434-49. doi: 10.1177/13591053221120968 [published Online First: 20220915]

57. Mehta S, Yarnell C, Shah S, et al. The impact of the COVID-19 pandemic on intensive care unit workers: a nationwide survey. Can J Anaesth 2022;69(4):472-84. doi: 10.1007/s12630-021-02175-z [published Online First: 20211223]

58. Işik M, Kirli U, Özdemir PG. The Mental Health of Healthcare Professionals During the COVID-19 Pandemic. Turk Psikiyatri Derg 2021;32(4):225-34. doi: 10.5080/u25827

59. Seifeldin Abdeen M, Mohammed MZ, El Hawary Y, et al. Traumatic stress in Egyptian doctors during COVID-19 pandemic. Psychol Health Med 2023;28(1):171-78. doi: 10.1080/13548506.2022.2059096 [published Online First: 20220330]

© 2024 Kamra M et al. *JAMA Network Open.*

60. Real-Ramírez J, García-Bello LA, Robles-García R, et al. Well-being status and post-traumatic stress symptoms in health workers attending mindfulness sessions during the early stage of the COVID-19 epidemic in Mexico. 2020 2020;43(6):8. doi: 10.17711/sm.0185-3325.2020.041 [published Online First: 2020-12-01]

© 2024 Kamra M et al. *JAMA Network Open*.
