# Supplementary material for: Physician Posttraumatic Stress Disorder During COVID-19: A Systematic Review and Meta-Analysis
Source: JAMA Netw Open. 2024 Jul 24;7(7):e2423316. doi: 10.1001/jamanetworkopen.2024.23316 (PMC11270139; doi:10.1001/jamanetworkopen.2024.23316)
Supplement: Supplement 2. — Data Sharing Statement [file jamanetwopen-e2423316-s002.pdf]

Data Sharing Statement

Kamra. Physician Posttraumatic Stress Disorder During COVID-19. *JAMA Netw Open*. Published July 19, 2024. doi:10.1001/jamanetworkopen.2024.23316

Data
Data available: No

Additional Information
Explanation for why data not available: All extracted and calculated data are available by emailing to the corresponding author on reasonable request.
